# FDA-Approved Drugs for Hematological Malignancies—The Last Decade Review

**DOI:** 10.3390/cancers14010087

**Published:** 2021-12-24

**Authors:** Aleksandra Sochacka-Ćwikła, Marcin Mączyński, Andrzej Regiec

**Affiliations:** Department of Organic Chemistry and Drug Technology, Faculty of Pharmacy, Wroclaw Medical University, 211A Borowska Street, 50-556 Wroclaw, Poland; marcin.maczynski@umw.edu.pl (M.M.); andrzej.regiec@umw.edu.pl (A.R.)

**Keywords:** small molecule agents, macromolecule agents, hematological malignancies, FDA, EMA

## Abstract

**Simple Summary:**

Hematological malignancies are diseases involving the abnormal production of blood cells. The aim of the study is to collect comprehensive information on new drugs used in the treatment of blood cancers which have introduced into therapy in the last decade. The approved drugs were analyzed for their structures and their biological activity mechanisms.

**Abstract:**

Hematological malignancies, also referred to as blood cancers, are a group of diseases involving abnormal cell growth and persisting in the blood, lymph nodes, or bone marrow. The development of new targeted therapies including small molecule inhibitors, monoclonal antibodies, bispecific T cell engagers, antibody-drug conjugates, recombinant immunotoxins, and, finally, Chimeric Antigen Receptor T (CAR-T) cells has improved the clinical outcomes for blood cancers. In this review, we summarized 52 drugs that were divided into small molecule and macromolecule agents, approved by the Food and Drug Administration (FDA) in the period between 2011 and 2021 for the treatment of hematological malignancies. Forty of them have also been approved by the European Medicines Agency (EMA). We analyzed the FDA-approved drugs by investigating both their structures and mechanisms of action. It should be emphasized that the number of targeted drugs was significantly higher (46 drugs) than chemotherapy agents (6 drugs). We highlight recent advances in the design of drugs that are used to treat hematological malignancies, which make them more effective and less toxic.

## 1. Introduction

Hematological malignancies, also known as blood cancers, are diseases characterized by the clonal proliferation of blood-forming cells, which occur in blood, bone marrow, or lymph nodes. Hematological malignancies include wild range types of leukemia, lymphoma, and myeloma, classified into two types: lymphoid and myeloid [1]. According to its mechanism of action, the drugs used for the treatment of hematological malignancies can historically be divided into the following groups: deoxyribonucleic acid (DNA)-interactive agents, antimetabolites, anti-tubulin agents, and molecular targeting agents such as highly specific small molecules and monoclonal antibodies. DNA interactive agents, the oldest group of anticancer medications, can be primarily categorized into alkylating agents, cross-linking agents, intercalating agents, topoisomerase inhibitors, and DNA-cleaving agents [2]. The first alkylating agent approved by the Food and Drug Administration (FDA) was **chlormethine** (**mechlorethamine**), also called nitrogen mustard. Goodman and coworkers described, in 1946, the pharmacological effect of **mechlorethamine** on Hodgkin’s lymphoma, lymphosarcoma, and leukemia [3], which led to this drug being registered in 1949 [4]. As a result of work on folic acid antagonists carried out by Farber, the next class of drug was developed, i.e., antifolate. In 1948, Farber reported the use of **aminopterin**, which was the 4-amino derivative of folic acid, to treat children with acute leukemia [5]. **Methotrexate** (**amethopterin**) replaced **aminopterin** in the treatment of patients in 1953 because it has a better therapy-versus-toxicity ratio [6,7]. Then, **mercaptopurine** and **fluorouracil** were discovered as the first structural analogs of purine and pyrimidine, respectively. **Mercaptopurine** was synthesized by Elion et al. in 1952 [8] and was first FDA-approved in 1953 [9], while **fluorouracil** was developed by Dushinsky et al. in 1957 [10] and received first approval in 1962 [11]. These drugs were widely used for the treatment of both solid and hematological malignancies [12]. Generally, folate, purine, and pyrimidine antagonists form one of the oldest classes of anticancer drugs, i.e., antimetabolites. The next discovered agents for the treatment of hematological malignancies were natural plant alkaloids with anti-tubulin activity. Noble and Beer isolated two first vinca alkaloids, i.e., **vinblastine** and **vincristine**, from *Catharanthus roseus* (L.) G. Don [13]. Both compounds received extensive clinical evaluation leading to the FDA approval of **vincristine** in 1963 as therapies for a variety of cancers [14]. Other natural products were cytotoxic antibiotics such as **bleomycin** and **doxorubicin**. **Bleomycin** was found in *Streptomyces verticillus* by Umezawa et al. in 1962. This antibiotic was the first DNA-cleaving agent to be registered, in 1973 [15], and can be used to treat malignant lymphoma as well as squamous cell carcinoma of the skin, head, and neck [16]. **Doxorubicin** was isolated from *Streptomyces peucetius* var. *caesius* in 1967 in Italy [17], and was first FDA-approved in 1974 [18]. The drug showed anticancer activity via multiple mechanisms including intercalation into DNA and inhibition of topoisomerase II activity. **Doxorubicin** was commonly used for the treatment of various hematological malignancies [19]. In 1965, Rosenberg and co-workers discovered that **cisplatin**, the platinum coordination complex synthesized by Peyrone for the first time in 1845 [20], caused inhibition of cellular division [21]. Then, **cisplatin** was entered in trials against a wide range of cancers where it showed potent anticancer activity through the cross-linking of DNA. The drug was approved by the FDA in 1978 and, since that time, has been used as a first-line treatment for patients with leukemia or lymphomas. Currently, it is still one of the most successful anticancer agents used in clinical practice [22]. A milestone for blood cancer treatment was the discovery of targeted therapy, consisting of the inhibition of molecular targets that are specific molecules involved in the growth, progression, and spread of cancer by monoclonal antibodies or small selective molecules. The first FDA-approved monoclonal antibody for the treatment of hematological malignancies, a genetically engineered chimeric anti-cluster of differentiation 20 (CD20) antibody, was **rituximab**. The drug was registered in 1997 for the treatment of relapsed or refractory, B-cell, low-grade, or follicular non-Hodgkin’s lymphoma (LG/F NHL) [23]. **Imatinib** was the first small molecule inhibitor (SMI) to be found to be selective against various protein tyrosine kinases. It was synthesized by Buchdunger in 1996 and approved by the FDA in 2001. The drug was indicated for patients with chronic myelogenous leukemia (CML) [24].

This article is an overview of drugs used in the treatment of hematological malignancies, which was approved by the FDA from 2011 until 2021. The most recent examples of small molecule and macromolecule drugs are detailed, focusing on the initial approval date, chemical structure, molecular target, route of administration, indication, and the most common adverse effects for each agent. Depending on the mechanism of action, the approved drugs are assigned to two categories: chemotherapy and targeted agents. In the present review, the medications containing a new molecular entity, or old active ingredient but in a new formulation, are summarized. The drugs referred to as biosimilars are also included. The biosimilars are an important group of biologic medicines which, although similar in structure, purity, and function to their reference products, with no meaningful differences in clinical efficacy and safety, increase access to hematologic malignancy therapies by mitigating the treatment costs [25]. Notably, the drugs received supplemental indications in the period from 2011 to 2021 but were originally approved before 2011, and drugs used to treat the side effects of cancer treatment are not included in this work.

## 2. Small Molecule Anticancer Drugs

### 2.1. Various Protein Kinase Inhibitors as Anticancer Agents

Protein kinases are enzymes, which catalyse the reversible phosphorylation of proteins. This reaction is one of the most important regulatory mechanisms and plays a crucial role in processes such as the transduction of external signals and the cell cycle regulation. Therefore, protein kinases inhibitors are an important group in need of new drugs, especially anticancer drugs. Protein kinase inhibitors are divided into three types. Type I inhibitors bind within and around the adenosine triphosphate (ATP) binding site of a catalytically active protein kinase, causing inhibition of its phosphorylation. Type II inhibitors bind to a hydrophobic pocket adjacent to the ATP binding site and are usually nonselective. In contrast, type III inhibitors bind to allosteric sites, remote from the ATP site, and are highly selective [26].

#### 2.1.1. Tyrosine Kinase (TK) Inhibitors

Tyrosine kinases (TKs) are enzymes that selectively phosphorylate the hydroxyl groups of a tyrosine residue in different proteins using ATP. They have a share in the regulation of most fundamental cellular processes such as growth, differentiation, proliferation, survival, migration, and the metabolism of cells, as well as programmed cell death in response to extracellular and intracellular stimuli [27]. The human genome contains at least 90 tyrosine kinase genes, which codify 58 receptor tyrosine kinases (RTKs) and 32 nonreceptor tyrosine kinases (NRTKs) [28]. RTKs are surface transmembrane receptors with kinase activity. In the structure of the receptor tyrosine kinases, an extracellular ligand-binding domain occurs which is connected to an intracellular catalytic kinase domain by a single pass transmembrane hydrophobic helix [27]. RTKs are not phosphorylated and monomeric in an inactive state [29]. Activation by ligand binding to their extracellular domain results in receptors’ oligomerization and autophosphorylation of a tyrosine residue within the kinase domain. NRTKs are cytoplasmic proteins that have a kinase domain and various additional signaling or protein-protein interacting domains [27]. They are activated by intracellular signals through the dissociation of inhibitors, by recruitment to transmembrane receptors, and through trans-phosphorylation by other kinases [29]. A large number of RTKs and NRTKs are associated with cancers; thus, a significant number of tyrosine kinase inhibitors (TKIs) are currently in clinical development. In the last 10 years, the FDA has approved four new drugs for the treatment of hematological malignancies, which are tyrosine kinase inhibitors (Table 1). Among them, there are the agents that target non-receptor Bruton’s tyrosine kinase (BTK) or non-receptor Sarcoma (Src) and Abelson (Abl) kinases. 

**Figure 1:**
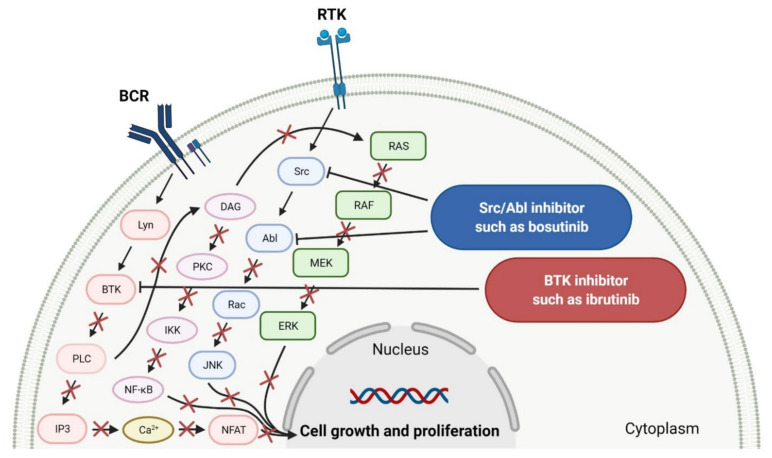
Mode of action of tyrosine kinase (TK) inhibitors such as non-receptor BTK and Src/Abl inhibitors. **BCR**: B-cell receptor. **RTK**: tyrosine kinase receptor. **RAF**: proto-oncogene serine/threonine-protein kinase. **MEK**: mitogen-activated protein kinase kinase. **ERK**: mitogen-activated protein kinase. **Src**: non-receptor Sarcoma kinase. Abl: Abelson kinase. **Rac**: Ras-related C3 botulinum toxin substrate. **JNK**: c-Jun N-terminal kinase. SYK: spleen tyrosine kinase. **BCAP**: B cell adapter for PI3K. **DAG**: diacylglycerol. **PKC**: protein kinase C. **IKK**: IκB kinase. **NF-κB**: nuclear factor kappa-light-chain-enhancer of activated B cells. **Lyn**: tyrosine-protein kinase Lyn. **BTK**: Bruton’s tyrosine kinase. **PLC**: phospholipase C. **IP3**: inositol trisphosphate. **NFAT**: nuclear factor of activated T-cells. Created with BioRender.com based on information in [37,38].

**Ibrutinib**, **acalabrutinib**, and **zanubrutinib** were originally developed as second-line therapy for the treatment of mantle cell lymphoma (MCL), a rare and aggressive type of blood cancer. To date, **ibrutinib** has received 11 FDA approvals since it was first registered in 2013, among others, as a breakthrough therapy for patients with Waldenström’s macroglobulinemia (WM) and chronic lymphocytic leukemia (CLL), who carry a deletion in chromosome 17 (17p deletion). In 2019, the FDA approved **ibrutinib** in combination with **obinutuzumab**, an anti-CD20 monoclonal antibody, as the first non-chemotherapy regimen for patients with previously untreated CLL [30]. In the same year, **acalabrutinib** received approval as the second BTK inhibitor to treat patients with CLL or small lymphocytic lymphoma (SLL). This drug can be used as monotherapy or in combination with **obinutuzumab** [31]. The mechanism of action of **ibrutinib**, **acalabrutinib**, and **zanubrutinib** is the irreversible inhibition of BTK activity by forming a covalent bond with a cysteine residue in the BTK active site. This results in blocking B cell antigen receptor signaling (i.e., nuclear factor of activated T-cells (NFAT) pathway, nuclear factor kappa-light-chain-enhancer of activated B cells (NF-κB) pathway, and mitogen-activated protein kinase (ERK) pathway), thus inhibiting the malignant B cells’ proliferation and survival (Figure 1) [32,33,34].

**Bosutinib** is a dual inhibitor of Src and Abl kinases that is used as a treatment for patients with Philadelphia chromosome positive (Ph+) chronic myeloid leukemia (CML), who show resistance or intolerance to previous therapy, including **imatinib**. The indication was extended in 2017 to include patients with newly diagnosed chronic phase Ph+ CML [35]. The drug shows activity against most imatinib-resistant mutants of BCR-ABL, which is a hybrid of a breakpoint cluster region protein (BCR) and Abelson tyrosine kinase (Abl), except the mutations T315I and V299. **Bosutinib** does not inhibit either the receptor tyrosine kinase c-Kit (known as mast/stem cell growth factor receptor or CD117) or platelet-derived growth factor receptor (PDGFR) [36]. The drug acts by binding to the active conformation of the kinase domain and inhibiting its autophosphorylation, resulting in a blockade of cancer cell growth (Figure 1) [37].

#### 2.1.2. Multi Kinase Inhibitors

**Figure 2:**
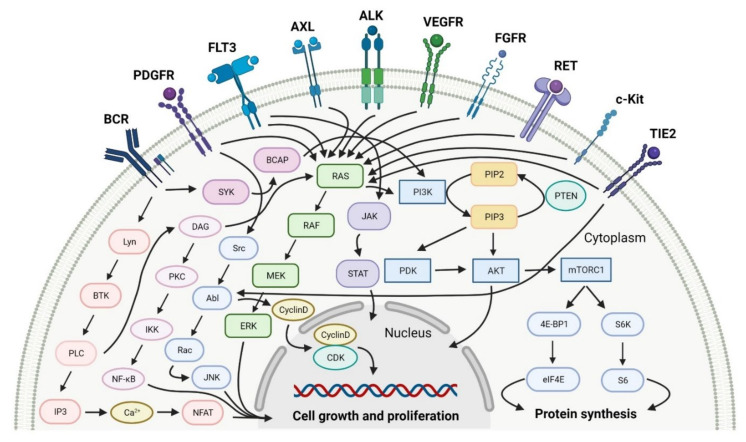
Schematic representation of the signaling pathways that can potentially be inhibited by multi kinase inhibitors. **BCR**: B-cell receptor. **PDGFR**: platelet-derived growth factor receptor. **FLT3**: FMS-like tyrosine kinase-3. **AXL**: AXL receptor tyrosine kinase. **ALK**: anaplastic lymphoma kinase. **VEGFR**: vascular endothelial growth factor receptor. **FGFR**: fibroblast growth factor receptor. **RET**: receptor tyrosine kinase rearranged during transfection. **c-Kit**: mast/stem cell growth factor receptor. **TIE2**: tunica interna endothelial cell kinase 2. **PI3K**: phosphatidylinositol 3-kinase. **PIP2**: phosphatidylinositol 4,5-bisphosphate. **PIP3**: phosphatidylinositol-3,4,5-trisphosphate. **PTEN**: phosphatase and tensin homolog deleted on chromosome ten. **PDK**: 3-phosphoinositide-dependent protein kinase. **AKT**: protein kinase B. mTORC1: mammalian target of rapamycin complex 1. **4E-BP1**: 4E-binding protein 1. **eIF4E**: eukaryotic translation initiation factor 4E. **S6K**: p70S6 kinase. **S6**: S6 protein. **RAF**: proto-oncogene serine/threonine-protein kinase. **MEK**: mitogen-activated protein kinase kinase. **ERK**: mitogen-activated protein kinase. **Src**: non-receptor Sarcoma kinase. **Abl**: Abelson kinase. **Rac**: Ras-related C3 botulinum toxin substrate. **JNK**: c-Jun N-terminal kinase. **CDK**: cyclin-dependent kinase. **SYK**: spleen tyrosine kinase. **BCAP**: B cell adapter for PI3K. **DAG**: diacylglycerol. **PKC**: protein kinase C. **IKK**: IκB kinase. **NF-κB**: nuclear factor kappa-light-chain-enhancer of activated B cells. **Lyn**: tyrosine-protein kinase Lyn. **BTK**: Bruton’s tyrosine kinase. **PLC**: phospholipase C. **IP3**: inositol trisphosphate. **NFAT**: nuclear factor of activated T-cells. Created with BioRender.com based on information in [37,38,50,51].

Multi kinase inhibitors are a group of ATP-competitive drugs that target a set of structurally related kinases. A single multi-inhibitor is preferred to two single inhibitors since drug-drug interactions might occur, changing the metabolism and activities against particular kinases. Multi kinase drugs become the second choice when their pharmacokinetic properties are worse. Besides, multi kinase inhibitors are less specific and might consequently lead to more side effects. A frequently observed disadvantage during treatment with multi kinase inhibitors is acquired resistance [48]. However, the inhibition of several kinases by one drug is useful in anticancer therapy, because oncogenesis and cancer growth have to be considered as multistep processes that are dependent on various signaling pathways (Figure 2) [49]. An overview of FDA-approved multi kinase inhibitors is presented in Table 2.

The kinase domain mutations are the reason for developing drug resistance during the treatment of various types of leukemia. One of the most common genetic alterations is the gatekeeper T315I substitution observed in chronic myeloid leukemia (CML) or Philadelphia chromosome positive (Ph+) acute lymphoblastic leukemia (ALL) and FMS-like tyrosine kinase-3 (FLT3)-activating mutations in acute myeloid leukemia (AML). Resistance to tyrosine kinase inhibitors has necessitated the designing of new mutation-resistant inhibitors, such as **ponatinib**, **midostaurin**, and **gilteritinib**. **Ponatinib** is a multitarget inhibitor characterized by high-affinity and optimized binding to the active site of the BCR-ABL kinase domain, in which the T315 can occur. This mutation is the major reason for inhibition access of the drug to the enzyme’s ATP-binding site, leading to resistance to first- and second-generation tyrosine kinase inhibitors [52]. **Ponatinib** is effective in the inhibition of native and mutant BCR-ABL, receptor tyrosine kinase rearranged during transfection (RET), FLT3, tunica interna endothelial cell kinase 2 (TIE2), mast/stem cell growth factor receptor (c-Kit), vascular endothelial growth factor receptors (VEGFRs), fibroblast growth factor receptors (FGFRs), and platelet-derived growth factor receptors (PDGFRs). Treatment with **ponatinib** shows substantial and durable clinical activity in patients with Ph+ leukemia with resistance or intolerance to all other approved tyrosine kinase inhibitors [53]. Adverse events of this therapy are defined as follows: nonhematologic toxic effects such as skin disorders (e.g., rash, acneiform dermatitis, and dry skin), constitutional symptoms (e.g., arthralgia, fatigue, and nausea), or hematologic—such as vascular occlusive—events, venous thromboembolic events, thrombocytopenia, and neutropenia [52]. The occurrence of vascular events during therapy was dependent on the dose of **ponatinib**, wherein lower doses have affected the improvement of the vascular safety profile [54]. **Midostaurin** and **gilteritinib** are approved drugs for patients with newly diagnosed FLT3-mutated AML. The clinical activity of **midostaurin** in combination with **cytarabine** and **daunorubicin**-based chemotherapy was positive for FLT3-activating mutations, such as, primarily, in-frame internal tandem duplications (ITD) and missense point mutations in the tyrosine kinase domain (TKD). Moreover, **midostaurin** inhibits c-Kit (wild type and D816V mutant) found in advanced systemic mastocytosis (SM), which includes aggressive systemic mastocytosis (ASM), systemic mastocytosis with associated hematological neoplasm (SM-AHN), and mast cell leukemia [55]. It was found to also be an inhibitor of protein kinase C (PKC), platelet-derived growth factor receptors (PDGFRs) alpha and beta, cyclin-dependent kinase 1 (CDK1), spleen tyrosine kinase (SYK), and vascular endothelial growth factor receptor-2 (VEGFR-2) [56]. Although **midostaurin** shows a broad spectrum of antikinase activity, it is characterized by lacked potency. **Gilteritinib**, on the other hand, is a selective, potent inhibitor of all FLT3-activating mutation types (e.g., ITC, TKD, D835Y, double ITD-D835Y) [57]. Furthermore, **gilteritinib** shows activity against c-Kit and the AXL receptor tyrosine kinase (AXL, also known as UFO), which is implicated in FLT3 inhibitor resistance [58]. The mechanism of action of **gilteritinib** involves binding to the active conformation of FLT3 at the ATP-binding site, resulting in reduced proliferation of cancer cells that overexpress the mutation [59].

The mutations that confer activation of the intracellular Janus kinase (JAK) signal transducer and activator of transcription (STAT) pathways (e.g., JAK2, V617F, and JAK2 exon 12) were identified as the most common in patients with myelofibrosis (MF). Only two drugs, namely **ruxolitinib** and **fedratinib**, are approved as JAK inhibitors for the treatment of MF [60]. **Ruxolitinib** is a JAK1/2 inhibitor that potently inhibits the proliferation of JAK2 V617F-driven Ba/F3 cells, resulting in decreased levels of phosphorylated JAK2 and signal transducer and activator of transcription 5 (STAT5) [61]. **Ruxolitinib** provides a rapid reduction in splenomegaly, ameliorating debilitating myelofibrosis-related symptoms and improving quality of life in patients with MF. The adverse events of **ruxolitinib**, like anemia and thrombocytopenia, were manageable and led to the discontinuation of therapy at a low rate [62]. **Ruxolitinib** is also an effective drug for patients with polycythemia vera, which allows for hematocrit control, reducing spleen size, and improving symptoms of disease [63]. However, some patients lose response to **ruxolitinib** and discontinue treatment over time because developing resistance or intolerance is associated with a substantially reduced life expectancy. **Fedratinib**, an alternative approved JAK inhibitor, is potent and selective for JAK2 regardless of its mutational status [64]. Compared with **ruxolitinib**, **fedratinib** causes a more effective reduction in spleen volume and disease-related symptoms [65].

### 2.2. Phosphatidylinositol 3-Kinase (PI3K) Inhibitors as Anticancer Agents

**Figure 3:**
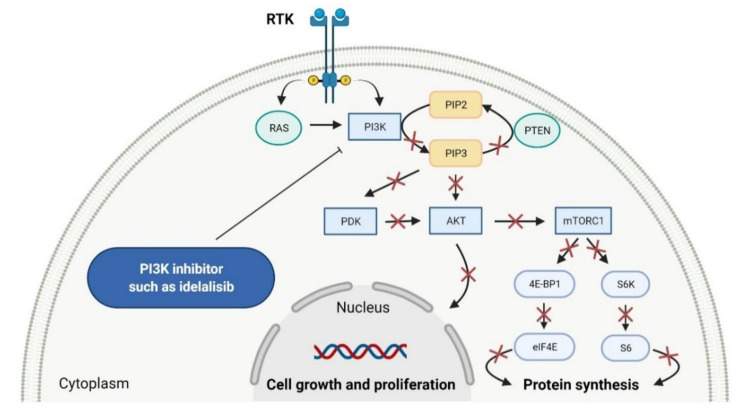
Mechanism of action of PI3K inhibitors. **RTK**: receptor tyrosine kinase. **PI3K**: phosphatidylinositol 3-kinase. **PIP2**: phosphatidylinositol 4,5-bisphosphate. **PIP3**: phosphatidylinositol-3,4,5-trisphosphate. **PTEN**: phosphatase and tensin homolog deleted on chromosome ten. **PDK**: 3-phosphoinositide-dependent protein kinase. **AKT**: protein kinase B. mTORC1: mammalian target of rapamycin complex 1. **4E-BP1**: 4E-binding protein 1. **eIF4E**: eukaryotic translation initiation factor 4E. **S6K**: p70S6 kinase. **S6**: S6 protein. Created with BioRender.com based on information in [51,79].

Phosphatidylinositol 3-kinases (PI3Ks) are a family of lipid kinases that phosphorylate phosphoinositides at the 3-hydroxyl group of the inositol ring that can be used to generate phosphatidylinositol 3,4,5-trisphosphate. Among these, several classes have been identified and characterized by different primary structures and substrate specificities. Class I PI3Ks are heterodimers and divide into two groups, IA and IB. Class IA PI3Ks are activated by a wide range of receptor tyrosine kinases (RTKs) and are frequently implicated in cancer, while class IB PI3Ks are activated by G-protein-coupled receptors [77]. Structurally, class IA PI3Ks exists in three isoforms (α, β, and δ) and class IB PI3Ks in one isoform (γ). Class II PI3Ks are monomeric proteins that consist of three isoforms (α, β, and δ), whereas class III PI3Ks are only one heterodimer composed of a catalytic (Vps34) and regulatory subunit [78]. PI3K-related kinases, which can be included as class IV of PI3K, are a group of protein kinases with structural similarity to PI3K, but without the lipid kinase activity. This group includes a mammalian target of rapamycin (mTOR), DNA-dependent protein kinase (DNA-PK), ataxia telangiectasia mutated gene product (ATM), and ataxia telangiectasia and Rad3-related gene product [79]. The dysregulation of the phosphatidylinositol-3 kinase pathway, especially abnormal activation, is one of the most frequently observed in blood cancers and an important target of selective anticancer therapies. The three inhibitors of PI3K have received market approval since 2011 (Table 3). **Idelalisib**, a first-in-class inhibitor of PI3K-δ, and the following inhibitors, i.e., **copanlisib** and **duvelisib**, directly reduce the proliferation and survival of malignant B-cell leukemia and lymphoma cells (Figure 3). Hence, they were approved by the FDA for the treatment of different types of leukemia and lymphoma [80,81,82]. **Duvelisib** is a first-in-class dual inhibitor of PI3Ks due to the fact that it also inhibits PI3K-γ activity, which leads to a reduction in the differentiation and migration of various components of the cancer microenvironment, such as T helper cells and M2 tumor-associated macrophages [82]. It is worth noting that the route of **copanlisib** administration as intermittent intravenous infusions lead to weaker gastrointestinal toxicity compared with the oral treatment of **idelalisib** [83].

### 2.3. Various Enzymes Inhibitors as Anticancer Agents

**Figure 4:**
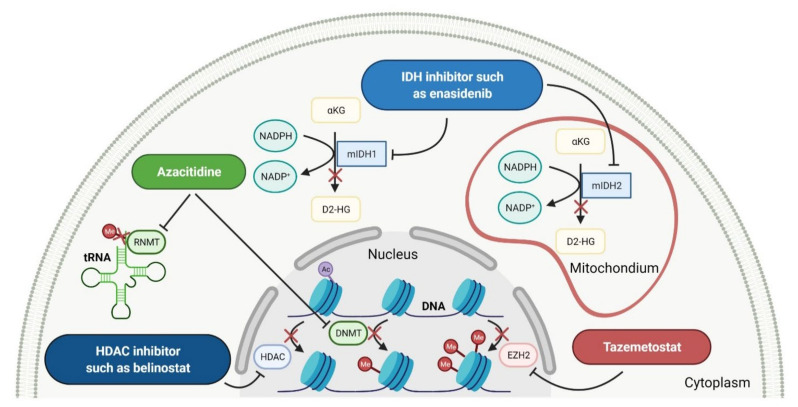
Mode of action of various enzymes inhibitors. **HDAC**: histone deacetylase. **RNMT**: RNA methyltransferase. **DNMT**: DNA methyltransferase. **EZH2**: enhancer of zeste homolog 2. **mIDH1**: mutant isocitrate dehydrogenase 1. **mIDH2**: mutant isocitrate dehydrogenase 2. **NADPH**: nicotinamide-adenine dinucleotide phosphate (reduced form). **NADP^+^**: nicotinamide-adenine dinucleotide phosphate. **αKG**: α-ketoglutarate. **D2-HG**: D-2-hydroxyglutarate. **Me**: methyl group. **Ac**: acetyl group. **DNA**: deoxyribonucleic acid. **tRNA**: transfer ribonucleic acid. Created with BioRender.com.

Enzymes are organic high-molecular-weight molecules that catalyze the synthesis or degradation reaction of a specific enzyme’s substrate. Enzyme inhibitors bind to the enzyme, resulting in disruption of the normal formation of an intermediate enzyme-substrate complex. Enzyme inhibitors, depending on their mechanism of action, cause the modification of enzyme’s activity in three ways, i.e., irreversible, competitive, and non-competitive [90]. The drugs that have received FDA approval for blood cancer treatment since 2011 inhibit the catalytic activity of such enzymes as histone deacetylase (HDAC), isocitrate dehydrogenases (IDHs), enhancers of zeste homolog 2 (EZH2), and deoxyribonucleic acid (DNA) or ribonucleic acid (RNA) methyltransferase (Figure 4). All new drugs are summarized in Table 4.

#### 2.3.1. Histone Deacetylase Inhibitors

Histone deacetylase (HDAC) enzymes catalyze the removal of an acetyl group from lysine residues of histone and non-histone (e.g., transcription factor p53) proteins. This process has a key role in modifying the structure of nucleosomes, the fundamental units of chromatin. Subsequently, the remodeling of chromatin form from open to closed is essential to the regulation of gene expression [91]. HDAC enzymes can be divided into four groups: class I (HDACs 1, 2, 3, and 8), class II a/b (HDACs 4, 5, 6, 7, 9, and 10), class III (sirtuin enzymes), and class IV (HDAC11). Class I, II, and IV HDACs are zinc-dependent enzymes [92]. The deregulation of HDACs activity has been reported in various cancer cell lines; therefore, their inhibition may be an attractive anticancer therapy. HDAC enzyme inhibitors (HDACi) induce rapid histone hyperacetylation and relaxation of chromatin, which results in cellular differentiation and apoptosis of cancer cells. Additionally, they promote cell cycle arrest, inhibit angiogenesis of cancer vasculature, and regulate host immune responses [91]. Since 2011, two HDACi are approved as a treatment for hematological malignancies, **belinostat**, for patients with relapsed or refractory peripheral T-cell lymphoma (PTCL), and **panobinostat**, for patients with recurrent multiple myeloma who have received at least two prior treatment regimens, including bortezomib and an immunomodulatory agent [93,94]. Both drugs are inhibitors of I, II, and IV HDAC isoforms. Their mechanism of action is the chelation of a zinc ion through the hydroxamate group region of their structure, resulting in the blocking of histone deacetylation [95].

#### 2.3.2. Isocitrate Dehydrogenase Inhibitors

Isocitrate dehydrogenases (IDHs) are oxidoreductases presented as one of three isoforms, i.e., IDH1, IDH2, and IDH3, which are involved in the tricarboxylic acid (TCA) cycle, also called the Krebs cycle [96]. IDH1 and IDH2 are similar, homodimeric enzymes that catalyze the reversible conversion of isocitrate to α-ketoglutarate (α-KG), liberating CO_2_ and reducing nicotinamide adenine dinucleotide phosphate (NADP^+^) to NADPH. Although IDH1 and IDH2 catalyze identical reactions, these enzymes are situated in different localizations, namely, IDH1 in the cytoplasm and peroxisomes, and IDH2 in the mitochondrial matrix. Both IDHs play important roles in many cellular processes, including modulating the response to glucose by regulating insulin secretion, the metabolism of glutamine or synthesis, and the metabolism of lipids. What is more, the enzymes show antioxidant activity related to the regulation of cellular redox status, resulting in protection against lipid peroxidation and oxidative DNA damage [97]. A third IDH enzyme, IDH3, is a heterotetrameric protein that catalyzes the first oxidative reaction of the TCA cycle, which is the decarboxylation of isocitrate to αKG with a generation of reduced form of nicotinamide adenine dinucleotide (NADH) from nicotinamide adenine dinucleotide (NAD^+^) [96]. IDH1 and IDH2 mutations are found in multiple tumors, including glioma and acute myeloid leukemia (AML). They occur in a single arginine residue, i.e., Arg132 of IDH1 and Arg172 of IDH2, in the active catalytic sites of the enzyme. Further, IDHs acquire a neomorphic activity, resulting in a reduction in αKG into the oncometabolic product, D-2-hydroxyglutarate (D2-HG). D2-HG competitively inhibits α-KG-dependent dioxygenases resulting in DNA and histone hypermethylation, which leads to a block of normal differentiation processes via epigenetic and metabolic mechanisms and promotes oncogenic transformation [98]. Since 2011, the first-in-class mutant IDH (mIDH) inhibitors, such as **enasidenib** and **ivosidenib**, have been FDA-approved for relapsed or refractory AML. **Enasidenib** targets both IDH2 R140 and R172 isoforms, while **ivosidenib** targets a variety of IDH1 R132 mutants (R132C, R132G, R132H, R132S, and R132L) [99,100]. The drugs suppress the production of oncometabolite D2-HG from α-KG by binding to the allosteric site of the mutant enzyme, thus preventing a conformational change in its structure and inhibiting gain-of-function enzymatic activity [101]. Both of these induce terminal differentiation of leukemic bone marrow blasts, which is responsible for the clinical response and efficacy of treatment [100,102].

#### 2.3.3. Enhancer of Zeste Homolog 2 Inhibitor

Enhancer of zeste homolog 2 (EZH2) is a histone methyltransferase that catalyzes trimethylation of histone H3 at Lys 27 (H3K27me3). EZH2 is also a catalytic component of polycomb repressive complex 2 (PRC2), which is a group of important epigenetic regulators of gene expression for regulating the differentiation of healthy cells. Mutation or overexpression of the EZH2 gene plays a critical role in the development of various cancers such as CRC, melanoma, ovarian cancer, and breast cancer. Dysregulation of EZH2 as a histone modifier causes the proliferation of cancer cells and promotes their survival and metastasis, resulting in invasion and progression of a malignant tumor. Moreover, EZH2 is involved in the regulation of immune cells (e.g., T cells, natural killer cells, dendritic cells, and macrophages), which are essential components in the cancer microenvironment [103]. **Tazemetostat** is a first-in-class, potent, and highly selective EZH2 inhibitor. The mechanism of its action is blocking EZH2 activity, thus the trimethylation of H3K27, which results in tumor regressions [104]. In clinical development, **tazemetostat** showed a favorable safety profile and responses in patients with either lymphoma, including both germinal center B-cell-like (GCB) and non-GCB subtypes of diffuse large B-cell lymphoma (DLBCL) [105], or advanced solid tumors such as epithelioid sarcoma [106]. The New Drug Application for **tazemetostat** as the treatment of relapsed or refractory follicular lymphoma (FL) was accepted by the FDA in June 2020 [107].

#### 2.3.4. DNA and RNA Methyltransferases Inhibitor

DNA and RNA methyltransferases (DNMTs and RNMTs) are a family of enzymes that catalyze the methylation of DNA and RNA, respectively. The five human DNMTs, i.e., DNMT1, DNMT2, DNMT3A, DNMT3B, and DNMT3L, play important role in epigenetic gene regulation, including transcriptional silencing and transcriptional activation, whereas RNMTs are implicated in RNA stability, splicing, and epigenetic mechanisms [108,109]. **Azacitidine** is a cytidine nucleoside analog that is phosphorylated in cell to its active form, i.e., azacitidine triphosphate, and is incorporated into DNA and RNA. Subsequently, **azacitidine** reduces DNA and RNA methylation by noncompetitive inhibition of DNA methyltransferases and RNA methyltransferases, respectively. The resulting hypomethylation of DNA is involved in the activation and expression of genes which regulate cancer-suppressing functions and cell differentiation. The reduction in RNA methylation leads to decreased RNA stability and decreased protein synthesis. **Azacitidine** shows, also, direct cytotoxicity to abnormal hematopoietic cells in the bone marrow [110]. **Azacitidine** was originally approved in 2004 as an intravenous or subcutaneous treatment of myelodysplastic syndromes [111]. However, in 2020, **ONUREG** (a novel oral formulation of **azacitidine** as film-coated tablets) was registered as continued treatment of adult patients in the first remission with acute myeloid leukemia (AML) [112]. **ONUREG** is the first drug to receive FDA approval as maintenance therapy. An oral **azacitidine** formulation facilitates the drug administration, reduces the potential side effects associated with subcutaneous injection, and allows for the expansion of treatment regimens by searching for alternative doses or combinations of therapies [113].

### 2.4. Smoothed Receptor Antagonists as Anticancer Agents

**Figure 5:**
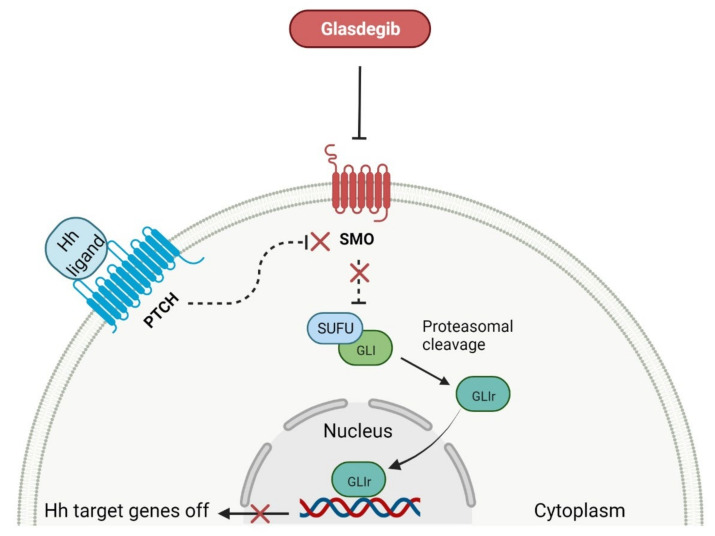
Glasdegib inhibition of the Hedgehog signaling pathway. **HH**: Hedgehog. **PTCH**: Patched receptor. **SMO**: smoothened receptor. **SUFU**: suppressor of fused protein. **GLI**: glioma-associated oncogene protein. **GLIr**: repressor form of GLI. Created with BioRender.com based on information in [128].

The deregulation of the smoothened (SMO) receptor’s activity is frequent in human cancers and has great biomedical importance for the development of new anti-cancer agents. The SMO receptor belongs to the class Frizzled (class F) receptors, which are part of a G-protein-coupled receptor (GPCR) family. SMO mediates signal transduction in the Hedgehog (Hh) pathway, which plays a significant role in normal embryonic development and maintenance or repair of adult tissue [124]. Under physiological conditions, the activation of the SMO receptor is regulated by the binding of hedgehog signaling proteins to a transmembrane receptor called Patched (PTCH), and leads to the downstream activation of the Hh signaling cascade. The abnormal Hh signaling is implicated in carcinogenesis, most notably in basal cell carcinoma (BCC) or medulloblastoma, and supports the tumor microenvironment in disparate cancers [125]. The SMO receptor activity can be modulated by small-molecule inhibitors, some of which are currently FDA-approved anticancer drugs, including **glasdegib**, **vismodegib**, and **sonidegib**. **Glasdegib**, as the only one that has been registered in the last 10 years, is characterized in Table 5. This drug potently inhibits the Hedgehog signaling pathway with clinical activity in older patients with newly diagnosed acute myeloid leukemia (AML) (Figure 5) [126]. It is used in combination with chemotherapy, i.e., **cytarabine**, which is an attractive treatment approach for patients with AML, improving statistically significant overall survival (OS) [127]. **Glasdegib** is the next generation SMO inhibitor, and does not use the same binding site as **vismodegib** and **sonidegib** [128]. Therefore, it is not susceptible to the evolution of drug resistances, especially by acquired mutations in SMO receptors. However, **vismodegib** and **sonidegib** are under intensive investigation in ongoing clinical trials as a therapy for blood cancer types such as myelofibrosis, chronic myeloid leukemia, and myelodysplastic syndromes.

### 2.5. Various Proteins Inhibitors as Anticancer Agents

**Figure 6:**
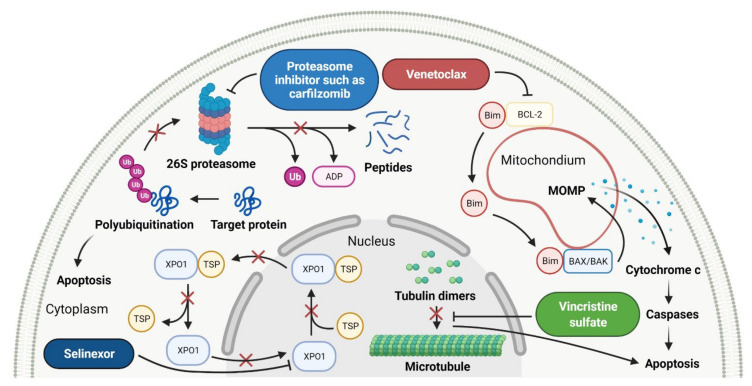
The four types of protein inhibitors in the treatment of hematological malignancies and their mechanisms of action. **BCL-2**: B-cell leukemia/lymphoma-2. **Bim**: BCL-2-like protein 11. **BAX**: BCL-2-associated X protein. **BAK**: BCL-2 antagonist/killer 1. **MOMP**: mitochondrial outer membrane permeabilization. **XPO1**: exportin-1. **TSP**: tumor suppressor protein. **Ub**: ubiquitin. **ADP**: adenosine diphosphate. Created with BioRender.com.

Proteins have a key role in an array of biological processes, such as signal transduction pathways or programmed cell death, and, therefore, offer an attractive target for anticancer therapy. In the last 10 years, there has been important progress in developing small-molecule inhibitors (SMIs) targeting various types of proteins, including B-cell leukemia/lymphoma-2 (BCL-2), exportin-1 (XPO1), proteasome, and tubulin protein (Figure 6). The features of the FDA-approved protein inhibitors are presented in Table 6.

#### 2.5.1. B-Cell Leukemia/Lymphoma-2 Proteins Inhibitor

Proteins of the B-cell leukemia/lymphoma-2 (BCL-2) family are key regulators of the mitochondrial apoptotic pathway. They control cell death primarily by forming pores within the mitochondrial outer membrane which results in the release of intermembrane space proteins (e.g., cytochrome c), followed by the activation of a caspase cascade and apoptosis. The BCL-2 family of proteins is divided into three main groups, including anti-apoptotic members (BCL-2, B-cell lymphoma-extra large (BCL-XL), BCL-W, myeloid cell leukemia 1 (MCL1)), the pro-apoptotic pore-formers (BCL-2-associated X protein (BAX), BCL-2 antagonist/killer 1 (BAK), BCL-2 related ovarian killer (BOK)), and the pro-apoptotic BH3-only proteins (BCL-2 associated agonist of cell death (BAD), BH3 interacting domain death agonist (BID), BCL-2 interacting killer (BIK), BCL-2-like protein 11 (Bim), BCL-2 modifying factor (BMF), Harakiri (HRK), NOXA, PUMA, etc.). All members of the BCL-2 family contain at least one of the four BCL-2 homologies (BH) domains (BH1–4), wherein the anti-apoptotic and pore-forming proteins are multi-BH domain molecules and BH3-only proteins possess only one helical BH3 domain [131,132]. The small molecules mimicking the BH3 domain of BH3-only proteins can selectively bind to and antagonize anti-apoptotic members of the BCL-2 family, leading to apoptosis of various malignancies cells. **Venetoclax** is a first-in-class, highly selective BCL-2 inhibitor that is bound to the BH3-binding groove of BCL-2 and releases pro-apoptotic BH3-only proteins (e.g., Bim) from BCL-2 [133]. Consequently, free BH3-only proteins activate pro-apoptotic effectors such as BAX and BAK that induce permeabilization of the mitochondrial outer membrane and trigger cell death. Furthermore, **venetoclax** also inhibits anti-apoptotic proteins such as MCL1. The drug received first approval as monotherapy in patients with chronic lymphocytic leukemia (CLL) with the 17p deletion who have received at least one prior therapy. The indication was extended on June 2018 to include use as a combination therapy with **rituximab** for CLL or small lymphocytic lymphoma (SLL), and on May 2019 to include use in combination with **obinutuzumab** for previously untreated patients with CLL or SLL. Finally, in October 2020, the FDA was granted full approval of **venetoclax** in combination with **azacitidine**, **decitabine**, or **cytarabine** for acute myeloid leukemia (AML) patients [134].

#### 2.5.2. Exportin-1 Inhibitors

Exportin-1 (XPO1), also known as chromosome maintenance protein 1 (CRM1), is a karyopherin involved in the export of various macromolecules from the nucleus to the cytosol, including tumor suppressor proteins (TSPs) (e.g., p53, p21, inhibitor of nuclear factor kappa B (IκB), p21), the glucocorticoid receptor, and messenger RNA (mRNA) for multiple oncoproteins (e.g., BCL-XL, mouse double minute 2 homolog (MDM2), cyclin D1). The export of mRNA to the cytoplasm is mediated exclusively by XPO1 after its binding to eukaryotic translation initiation factor 4E (eIF4e), which promotes the synthesis of cognate oncoproteins, cell survival, and proliferation [135]. Due to XPO1 being overexpressed in most cancer types, it is a promising target for anticancer therapy. **Selinexor** is a first-in-class selective inhibitor of nuclear export (SINE) compound. The drug forms a slowly reversible covalent bond to Cys528 in the cargo-binding pocket of XPO1, leading to inactivation of XPO1-mediated nuclear export [136]. The inhibition of XPO1 activity results in the accumulation of TSPs in the cell nucleus and the prevention of oncoprotein mRNA translation. It consequently leads to the induction of cell-cycle arrest and apoptosis [137]. **Selinexor** received first approval in combination with dexamethasone for the treatment of adult patients with relapsed or refractory multiple myeloma (MM), after at least four prior therapies. In June 2020, **selinexor** was registered, as well, for the treatment of adults with relapsed or refractory diffuse large B-cell lymphoma (DLBCL), who have previously attempted at least two lines of systemic therapy. The indication was then extended in December 2020 to include use in combination with **bortezomib** and **dexamethasone** for the treatment of adult patients with MM after at least one prior therapy [138].

#### 2.5.3. Proteasome Inhibitors

The proteasome is a large protein complex responsible for the selective and efficient hydrolysis of proteins. The degradation of protein is initiated by the covalent attachment of chain consisting of several copies of ubiquitin (Ub), this is a process known as protein ubiquitylation. The polyubiquitin (poly-Ub) chain, the most abundant Lys48-linked poly-Ub (Ub^Lys48^) chain, is an intracellular signal to shuttle the target proteins to the proteasome, where they are proteolytically broken down [139]. The constitutive 26S proteasome, the main extralysosomal mediator of protein degradation, consists of two protein-recognizing 19S regulatory units and a 20S proteolytic core. The 20S core particle includes three protein-specific catalytic subunits, i.e., β1, β2, and β5, that are referred to as caspase-like, trypsin-like, and chymotrypsin-like, respectively [140]. The proteasome plays a key role in controlling the levels of disparate regulatory proteins and prevents the accumulation of mutant or damaged proteins in the cell. Moreover, proteasome activity exerts influence on the other cellular process, including proliferation, cell death, signal transduction, immune response or metabolism [141]. The malignant plasma cells, in particular, are degraded by the ubiquitin-proteasome pathway; thus, they are susceptible to the action of proteasome inhibitors (PI), which are a mainstay in the treatment of multiple myeloma (MM). **Carfilzomib** and **ixazomib citrate** are highly effective in the second-generation of PI registered in combination with **dexamethasone** and immunomodulatory drugs (**lenalidomide**) for patients with MM, who have received at least one previous therapy [142,143]. **Carfilzomib** is also used as monotherapy and in combination with **dexamethasone** and monoclonal antibody (**daratumumab**) [143]. **Ixazomib citrate** is the first orally administrated PI which contains a citrate-protected boric acid group. The drug hydrolyzes under physiological conditions (e.g., in the gastrointestinal tract and plasma) to its biologically active metabolite, i.e., **ixazomib**. The mechanism of action of **carfilzomib** and **ixazomib** is forming irreversible and reversible, respectively, adducts with a 20S unit of 26S proteasome, mainly in the chymotrypsin-like site. At high concentrations, both drugs show additional inhibitory effects on the trypsin-like and caspase-like sites [144]. Due to proteasome inhibition, the drugs cause an increase in ubiquitinated proteins in MM cells, resulting in activation of an unfolded protein stress response and, ultimately, cell-cycle arrest and apoptosis. Programmed cell death induced by **carfilzomib** is associated with the activation of caspase pathways and c-Jun-N-terminal kinase (JNK). Moreover, **carfilzomib** promotes mitochondrial membrane depolarization and the release of cytochrome c [145]. The anti-myeloma effect induced by **ixazomib** is mainly related to the blocking of the NF-κB pathway, which reduces the release of cytokines secreted by bone marrow stream cells, important for the proliferation of MM cells. This drug also causes activation of caspase pathways, an increase in cleavage of poly (ADPribose) polymerase, the overexpression of a tumor suppressor gene miR33b, and reduced angiogenesis by blocking expression of the VEGFR-2 and platelet endothelial cell adhesion molecule (PECAM). Furthermore, the use of **ixazomib** leads to upregulation of signaling pathways such as p53-p21, p53-NOXA-PUMA, and E2 factor (E2F) [146].

#### 2.5.4. Tubulin Inhibitor

Tubulin is a protein which is essential for the normal polymerization of mitotic spindle microtubules, which play a pivotal role in cellular processes, including chromosome segregation during mitosis or the intracellular transport of organelles. Microtubules are also components of the cytoskeleton, determining cell shape. Structurally, they can be defined as cylindrical protofilaments composed of α/β-tubulin dimers. Microtubule-targeting agents (MTAs), e.g., **vincristine** (**VCR**), bind to tubulin proteins and, thus, inhibit their polymerization into microtubules. The effects of **VCR** on tubulin polymerization are dependent on the concentration of the dose. At low concentrations, **VCR** inhibits the formation of microtubules through the direct binding at α/β-tubulin dimers and indirect cross-linking of proteins which are responsible for the stabilization of protofilaments. This inhibition prevents chromosome segregation and results in arrest mitosis at the metaphase. At higher concentrations, **VCR** promotes the disruption and depolymerization of microtubules via binding tubules in a different conformation, leading to the formation of spiral aggregates and highly structured crystals. The alterations of the structure of the microtubules cause mitotic spindle disintegration and the accumulation of chromosomes, thus the activation of apoptotic pathways and cell death. Another mechanism of action of **VCR** includes the inhibition of axon transport, secretion processes, and impairment of platelet functions [147]. **Vincristine** is a vinca alkaloid that received initial approval in 1963, and, since then, has been used to treat different cancer types including leukemia, Hodgkin’s lymphoma, lung cancer, and breast cancer [14]. **Vincristine sulfate** encapsulated in sphingomyelin and cholesterol-based nanoparticles (**VSLI**; **MARQIBO**) is a novel formulation of **VCR** to treat adults with a rare type of leukemia called Philadelphia chromosome-negative (Ph-) acute lymphoblastic leukemia (ALL). **VSLI** consists of a uniquely slow-release liposomal system suited to deliver and intensify the dose of **VCR**. The liposomal carrier prolongs the circulation time of the drug, optimizes delivery to target tissues, and increases the therapeutic index. The long exposure time of cancer cells to **VSLI** leads to its therapeutic efficacy compared to standard **VCR**. These characteristics result in overcoming the dose-related neurotoxicity and pharmacokinetic limitations of free **VCR** [148]. 

### 2.6. Protein Translation Inhibitors as Anticancer Agents

**Figure 7:**
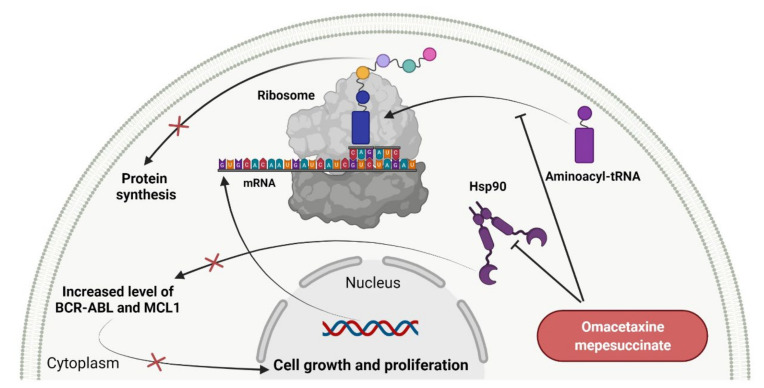
Schematic representation of major mechanisms of action for omacetaxine mepesuccinate. **BCR-ABL**: BCR-ABL fusion protein. **MCL1**: myeloid cell leukemia 1. **mRNA**: messenger ribonucleic acid. **tRNA**: transfer ribonucleic acid. **Hsp90**: heat shock protein 90. Created with BioRender.com.

Protein synthesis is the fundamental cellular process for living organisms, which can be defined as the transmitting of genetic information from DNA to proteins. First, the genetic code in the DNA is transcribed into messenger RNA (mRNA) in the nucleus. Next, information carried by the nucleotide sequence of mRNA is translated into the amino acid sequence of a polypeptide chain. The decoding of mRNA is performed after the transport of mRNA into the cytoplasm, where it becomes bound to the ribosome, and the placement of subsequent amino acid attached to transfer RNA (tRNA), i.e., aminoacyl-tRNA, in the A-site cleft of the ribosome. Finally, ribozyme, an integral part of the ribosome, catalyzes the formation of a peptide bond between amino acids [160]. Since components of protein translation machinery are often deregulated in cancer, inhibitors of protein synthesis are a promising group of anticancer drugs. **Omacetaxine mepesuccinate** is the first analog of cephalotaxine that has been approved by FDA for patients with chronic myeloid leukemia (CML) and who have demonstrated resistance or intolerance to two or more tyrosine kinase inhibitors (TKIs). The approval allows the home administration of subcutaneous injection using syringes prepared by a professional healthcare practicioner [161]. The drug is semisynthetic purified homoharringtonine, a natural alkaloid originally isolated from various *Cephalotaxus* species. The synthesis of **omacetaxine mepesuccinate** is based on direct esterification of cephalotaxine isolated from *Cephalotaxus* conifers [162]. **Omacetaxine mepesuccinate** acts by inhibiting protein synthesis during the G1 and G2 phases of the cell cycle (Figure 7). Its mechanism of its action is distinct from TKIs and includes binding to the A-site cleft of ribosomes that prevents the binding of incoming aminoacyl-tRNA to the ribosomal acceptor site and blocks peptide bond formation. In vitro studies have revealed that **omacetaxine mepesuccinate** selectively reduces levels of pro-oncogenic or anti-apoptotic proteins essential for leukemia cell survival, including both native and mutated forms of BCR-ABL and MCL1. The use of this drug leads to the degradation of BCR-ABL proteins by inhibiting heat shock protein 90 in a dose-dependent manner. The mutant BCR-ABL was more sensitive to inhibition by treatment with the drug compared to the native form of BCR-ABL. The results from in vitro studies have also demonstrated apoptotic activity of **omacetaxine mepesuccinate** related to activating caspase-9, caspase-8, caspase-3, and poly (ADP-ribose) polymerase (PARP) [163]. The overall characteristic of the drug is presented in Table 7.

### 2.7. Purine Antagonist as Anticancer Agent

**Figure 8:**
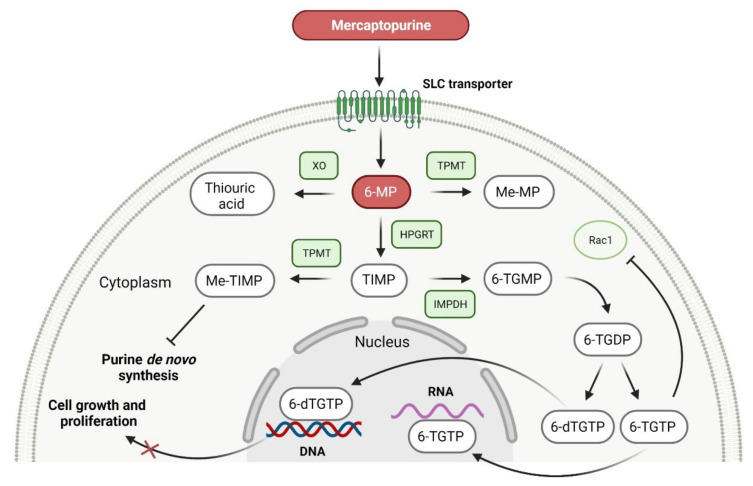
Scheme of metabolism and mode of action of mercaptopurine (6-MP). **SLC**: solute carrier. **6-MP**: mercaptopurine. **XO**: xanthine oxidase. **TPMT**: thiopurine methyltransferase. **Me-MP**: methylmercaptopurine. **HPGRT**: hypoxanthine-guanine phosphoribosyltransferase. **TIMP**: thioinosine monophosphate. Me-TIMP: methyl thioinosine monophosphate. **IMPDH**: inosine monophosphate dehydrogenase. **6-TGMP**: 6-thioguanosine monophosphate. **6-TGDP**: 6-thioguanosine diphosphate. **6-dTGTP**: deoxy-6-thioguanine triphosphate. **6-TGTP**: 6-thioguanosine triphosphate. **Rac1**: ras-related C3 botulinum toxin substrate 1. **DNA**: deoxyribonucleic acid. **RNA**: ribonucleic acid. Created with BioRender.com based on information in [167].

Purine antagonists, such as **mercaptopurine** (**6-MP**), belong to the class of drugs known as antimetabolites, which inhibit nucleic acid metabolism and are widely used in cancer chemotherapy. **Mercaptopurine** is a purine analog evaluated for the treatment of acute leukemias that act as antagonists to the endogenous purines [166]. It is a prodrug activated by hypoxanthine-guanine phosphoribosyltransferase (HGPRT), which converts it to thioinosine monophosphate (TIMP). TIMP is further methylated to 6-methylthioinosine monophosphate (Me-TIMP), a potent inhibitor of the purine de novo synthesis (Figure 8) [167]. Me-TIMP also reduces the amount of natural purines in lymphoblast, which results in the inhibition of DNA synthesis and early cytotoxicity in the S phase [168]. In addition, TIMP is metabolized to deoxy-6-thioguanine triphosphate (6-dTGTP) and 6-thioguanine triphosphate (6-TGTP), which are incorporated into DNA and RNA, respectively. This process causes inhibition of nucleotide and protein synthesis, thus inducing lymphocyte proliferation. 6-TGTP also inhibits Rac1, leading to apoptosis of T-lymphocytes via modulation of the Vav-Rac1 signaling pathway [169]. **Mercaptopurine** was approved for medical use in 1953 and was commercially available only as a 50 mg tablet until 2014, when the FDA approved a new liquid formulation of **6-MP** (**PURIXAN**) [170]. **PURIXAN** is a 20 mg/mL oral suspension indicated for the treatment of patients with acute lymphoblastic leukemia (ALL) as part of a combination regimen. This drug provides flexibility and precision in terms of dosing based on the individual patient’s dosing needs. Especially, **PURIXAN** improves dose adjustments and accurately delivers the desired dose for children with a wide range of weights. Moreover, suspension as a route of administration is more convenient than tablets [171]. The overall features of this drug are presented in Table 8.

### 2.8. Immunomodulators as Anticancer Agents

Immunomodulatory drugs (IMiDs) are a group of compounds that are structural and functional analogs of thalidomide, showing immunomodulatory, anti-angiogenic, and anti-inflammatory properties. IMiDs are currently under investigation and demonstrate activity against various hematologic malignancies, including multiple myeloma (MM), acute myeloid leukemia (AML), chronic lymphocytic leukemia (CLL), and myelofibrosis (MF), or solid tumors including lung, breast, renal, and colon cancer [174]. **Pomalidomide**, the newest IMiD characterized in Table 9, received approval in February 2013 as treatment for patients with multiple myeloma (MM) whose disease progressed after previous lines of therapy, including **lenalidomide** and **bortezomib**. On May 2020, the indication for treatment with **pomalidomide** was extended for patients with AIDS-related Kaposi sarcoma resistant to highly active antiretroviral therapy (HAART), or patients with HIV-negative Kaposi sarcoma [175]. **Pomalidomide’s** mechanism of action includes various molecular and cellular elements within the bone marrow microenvironment or cytokine production, as well as immunomodulation and direct antimyeloma activity. **Pomalidomide** causes a reduction in different proinflammatory cytokines, including tumor necrosis factor-alpha (TNF-α), interleukin-1β (IL-1β), and interleukin-6 (IL-6). Furthermore, the anti-inflammatory effects of the drug are related to the inhibition of cyclooxygenase-2 (COX-2) and prostaglandin production by monocytes. Immunomodulatory mechanisms include enhanced T-cell proliferation leading to the increased production of interleukin-2 (IL-2) and interferon-γ (IFN-γ), in addition to amplified natural killer (NK) cells activity and antibody-dependent cell-mediated cytotoxicity (ADCC) in MM cells [176]. **Pomalidomide** also reduces the IL-2-mediated generation of T regulatory cells (FOXP3^+^CTLA-4^+^CD4^+^CD25^high^ cells), which are important controllers of the immune response and suppressors of anti-tumor immunity [177]. Moreover, the drug shows antiangiogenic activity by inhibiting the secretion of vascular endothelial growth factor (VEGF) and hypoxia-inducible factor 1 alpha (HIF-1α). **Pomalidomide** achieves its antimyeloma action by causing both cell-cycle arrest and apoptosis. Specifically, the drug induces cell-cycle arrest via a Lysine-specific demethylase 1 (LSD1)-mediated epigenetic mechanism, resulting in p21 (WAF-1) activation independently of p53 signaling, apoptosis by activation of the caspase-8 pathway, increased sensitivity to Fas-mediated cell death, suppression of nuclear factor kappa-B (NF-κB) transcription, and inhibition of apoptosis 2 (IAP-2) [176]. The molecular target for **pomalidomide** is a highly conserved protein, cereblon (CRBN). The drug binds endogenous CRBN, resulting in the inhibition of its autoubiquitination and subsequent suppression of interferon regulatory factor 4 (IRF4), which is critical for MM cell survival [178]. Finally, clinical trials have proved that **pomalidomide** is the most potent IMiD [176].

### 2.9. Fixed-Dose Combination Drugs as Anticancer Agents

A fixed-dose combination (FDC) drug is a combination of two or more active agents within a single form of pharmaceutical administration. FDC drugs are a promising treatment strategy for anticancer therapy, due to that all of their ingredients reach a target cancer cell simultaneously and act by different mechanisms. The disruption of the cell cycle in different phases and the inhibition of various survival pathways results in better suppression of cancer chemoresistance, thereby synergistically inducing cellular apoptosis and inhibiting the growth of cancerous tissue. FDC drugs improve therapeutic efficiency and overcome potential adverse effects of treatment, as well as reduce the cost to patients compared to single drug therapies [181]. FDC drugs approved by the FDA since 2011 for treatment of certain hematological malignancies are shown in Table 10.

**VYXEOS** is the first dual-drug liposomal encapsulation product registered by the FDA. It is composed of **cytarabine** and **daunorubicin**, in a molar ratio of 5:1, which are entrapped in liposomes. The liposome membrane, which contained distearoyl phosphatidylcholine, distearoyl phosphatidylglycerol, and cholesterol in a molar ratio of 7:2:1, is degraded upon internalization of the drug into target cells. **Cytarabine** and **daunorubicin** are then released within the intracellular environment [182]. **Cytarabine**, a pyrimidine nucleoside analog originally approved in 1969, is incorporated into DNA resulting in inhibition of DNA synthesis in the S-phase of the cell cycle and subsequent apoptotic cell death [183,184]. **Daunorubicin**, an anthracycline antibiotic approved for medical use in 1979, induces inhibition of DNA synthesis by the intercalation and blocking of activity of topoisomerase II [185,186]. This drug also generates free radicals, leading to DNA damage. Both chemotherapeutic agents in the indicated proportion have synergistic effects to induce leukemia cell death. **VYXEOS** has demonstrated a clinically significant improvement in overall survival compared with conventional **cytarabine** plus **daunorubicin** chemotherapy (7 + 3 regimen) in adults with newly diagnosed secondary acute myeloid leukemia (sAML) and therapy-related acute myeloid leukemia (t-AML), as well as AML with myelodysplasia-related changes (AML-MRC) [187,188]. By the end of 2020, the drug received approval as a treatment for adult patients with newly diagnosed t-AML or AML-MRC [189].

**INQOVI** is a fixed-dose combination of **decitabine** and **cedazuridine**. In 2006, **decitabine** was approved as a single drug that had to be administered by intravenous injection because of its degradation by cytidine deaminase in the gastrointestinal tract and liver [190]. **INQOVI** is designed to enable oral delivery of **decitabine**, due to the inhibition of cytidine deaminase activity by **cedazuridine**. The mechanism of action of **decitabine**, a cytosine analog, includes incorporation into replicating DNA and the inactivation of DNA methyltransferases (DNMTs). The subsequent hypomethylation of DNA is responsible for cancer cell death through a variety of mechanisms [191]. **INQOVI** is indicated for the treatment of adult patients with newly diagnosed and secondary myelodysplastic syndromes (MDS), a group of bone marrow diseases that change the production of functional blood cells, including refractory anemia, refractory anemia with ringed sideroblasts, refractory anemia with excess blasts, and chronic myelomonocytic leukemia (CMML) [192].

## 3. Macromolecule Anticancer Drugs

### 3.1. Monoclonal Antibodies as Anticancer Agents

**Figure 9:**
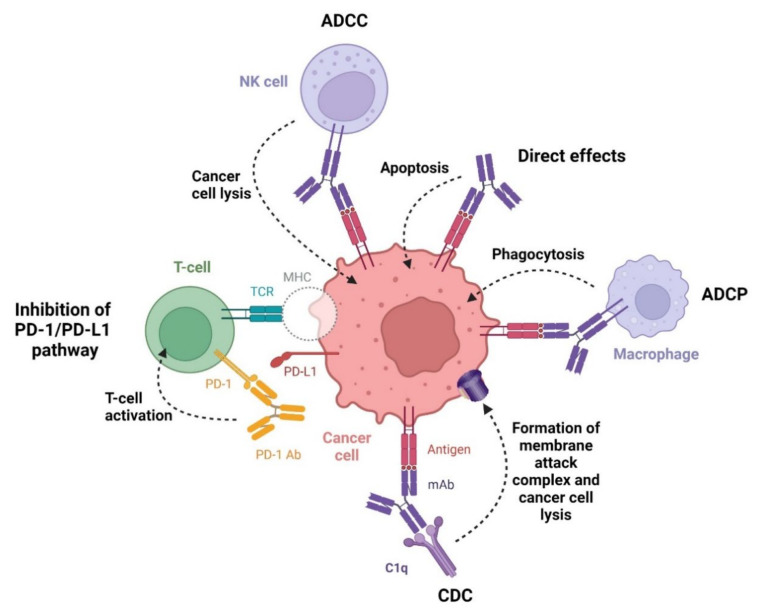
Mode of action of monoclonal antibodies. **ADCC**: antibody-dependent cellular cytotoxicity. **NK**: natural killer. **ADCP**: antibody-dependent cellular phagocytosis. **CDC**: complement-dependent cytotoxicity. **mAb**: monoclonal antibody. **C1q**: complement component 1q. **PD-1**: programmed death-1 protein. **PD-L1**: programmed death ligand-1. **PD-1 Ab**: monoclonal antibody directed against PD-1. **TCR**: T-cell receptor. **MHC**: major histocompatibility complex. Created with BioRender.com based on information in [197].

Monoclonal antibodies (mAbs) represent a clonal version of a specified antibody isotype that can bind to antigens found on the surface of cancer cells. The activity of mAbs leads to normalizing growth rates and induces cancer cells’ death via various mechanisms (Figure 9). Furthermore, mAbs target the cancer microenvironment and inhibit processes such as angiogenesis. Monoclonal antibodies are composed of two heavy and two light chains, which form three functional protein domains: two identical fragments for antigen binding (Fab regions) and the constant or crystallizable fragment (Fc region). Depending on the type of Fab regions, mAbs can be divided into four classes: murine, chimeric, humanized, and human [196]. Immunoglobulin-type (IgG-type) monoclonal antibodies, an important class of therapeutic proteins which are the most frequently used for cancer immunotherapy, are classified into immunoglobulin G1 (IgG1), immunoglobulin G2 (IgG2), immunoglobulin G3 (IgG3), and immunoglobulin G4 (IgG4) [197]. Monoclonal antibodies approved by the FDA in the last decade for the treatment of hematological malignancies target cluster of differentiation 19 (CD19), cluster of differentiation 20 (CD20), cluster of differentiation 38 (CD38), signaling lymphocyte activation molecule family member 7 (SLAMF7), and programmed death receptor-1 (PD-1). The registered mAb drugs are summarized in Table 11.

#### 3.1.1. Anti-CD19 Monoclonal Antibody

Cluster of differentiation 19 (CD19) is a glycoprotein with a single transmembrane domain specifically expressed in B lymphocytes. CD19 is a cell surface response regulator that modulates B cell signaling. It plays a role in the development and immunoglobulin-induced activation of B cells [198]. **Tafasitamab-cxix** is an Fc-modified humanized monoclonal antibody that binds to the CD19 antigen expressed on the surface of B-cell malignancies, including diffuse large B-cell lymphoma (DLBCL). The drug induces B-cell lysis via apoptosis and immune effector mechanisms, i.e., antibody-dependent cellular cytotoxicity (ADCC) and antibody-dependent cellular phagocytosis (ADCP). In the structure of the Fc region of **tafasitamab-cxix**, two amino acids were substituted, resulting in increased Fcγ receptor affinity compared with a non-engineered anti-CD19 mAbs [199]. The drug is used in combination with **lenalidomide** as a treatment for adult patients with relapsed or refractory DLBCL, who are not eligible for autologous stem cell transplant (ASCT) [200]. 

#### 3.1.2. Anti-CD20 Monoclonal Antibodies

Cluster of differentiation 20 (CD20) is a molecule with multiple membranes spanning domains expressed exclusively by B lymphocytes. The engagement of CD20 on antigen-presenting cells leads to the regulation of transmembrane transport of Ca^2+^ and cell-cycle progression during B cell activation and proliferation. Many hematological malignancies, including non-Hodgkin’s lymphomas, have been shown to express CD20; thus, anti-CD20 mAbs are an effective anticancer immunotherapy. Anti-CD20 mAbs bind to different epitopes on CD20, leading to the inhibition of B lymphocyte progression into the S/G2+M stages of the cell cycle [201]. There are two types of anti-CD20 mAbs based on different mechanisms causing malignant B cells death. Type I anti-CD20 antibodies, such as **rituximab** or its biosimilars **rituximab-abbs**, **rituximab-pvvr**, or **rituximab-arrx**, redistribute CD20 molecules into large insoluble lipid microdomains (lipid rafts) and exert potent complement-dependent cytotoxicity (CDC) activity. In contrast, type II antibodies such as **obinutuzumab** induce less accumulation of CD20 in lipid rafts and show weak CDC activity [202]. **Obinutuzumab** shows an increased ability to bind and recruit effector cells, thereby antibody-dependent cellular cytotoxicity (ADCC) activity, due to the glycoengineered the Fc region [203]. **Obinutuzumab** was more effective than **rituximab** as either first- or second-line therapy in mouse xenograft models of human lymphoma [204]. **Rituximab-abbs** received approval for the treatment of non-Hodgkin’s lymphoma (NHL), to be used as a single agent or in combination with chemotherapy, while **rituximab-pvvr** and **rituximab-arrx** were approved as single agents or in combination with first line chemotherapy to treat patients with NHL and chronic lymphocytic leukemia (CLL) [205,206,207]. **Obinutuzumab** was approved in combination with **chlorambucil** as a treatment for CLL and combination with **bendamustine** as a treatment for patients with follicular lymphoma (a type of non-Hodgkin lymphoma). This drug is also registered in combination with **ibrutinib** as a first non-chemotherapy treatment of patients with CLL [208].

#### 3.1.3. Anti-CD38 Monoclonal Antibodies

A cluster of differentiation 38 (CD38) is a transmembrane glycoprotein expressed extensively on B and T lymphocytes. It shows an aplysia adenosine diphosphate (ADP)-ribosyl cyclase activity, and the ability to regulate intracellular Ca^2+^ release [209]. Anti-CD38 mAbs represent a promising therapy for patients with multiple myeloma (MM) since CD38 is expressed at relatively low levels on myeloid and lymphoid cells, and at high levels in various malignant plasma cells, including MM. **Daratumumab** and **isatuximab**, FDA-approved anti-CD38 antibodies, kill MM cancer cells via Fc-dependent immune effector mechanisms, i.e., complement-dependent cytotoxicity (CDC), antibody-dependent cell-mediated cytotoxicity (ADCC), antibody-dependent cellular phagocytosis (ADCP), and apoptosis. Both drugs also show indirect anticancer activity consisting of the suppression of CD38+ T regulatory cells, thus restoring immune effector functions against MM [210,211]. On the other hand, **daratumumab** and **isatuximab** target a different amino acid sequence in CD38 molecules and exhibit several mechanistic differences. **Daratumumab** immediately induces redistribution of CD38 on the surface of MM cells and the formation of polar aggregates leading to the release of CD38 in microvesicles, while **isatuximab** does not result in a decrease in the CD38 level. **Isatuximab** induces direct apoptosis through the caspase-dependent apoptotic pathway and the lysosome-mediated cell death pathway, whereas **daratumumab** induces apoptosis only in the presence of cross-linking agents. **Isatuximab** strongly inhibits CD38 enzymatic activity, acting as an allosteric antagonist, while **daratumumab**, under the same experimental conditions, shows a more limited inhibition. Finally, **isatuximab** can regulate the cancer immunosuppressive environment of the bone marrow niche in MM patients via the modulation of adenosine levels [212]. **Daratumumab** was approved as monotherapy and in combination with various chemotherapy regiments (i.e., **lenalidomide** and **dexamethasone**, **bortezomib**, **melphalan** and **prednisone**, **bortezomib**, **thalidomide** and **dexamethasone**, **bortezomib** and **dexamethasone**, **pomalidomide** and **dexamethasone**, or **carfilzomib** and **dexamethasone**) to treat both relapsed or refractory multiple myeloma (RRMM) and newly diagnosed multiple myeloma (NDMM) [213]. **Isatuximab** was registered indicated with **pomalidomide** and **dexamethasone** for the treatment of adult patients with MM who have received at least  two prior therapies and in combination with **carfilzomib** and **dexamethasone** to treat adult patients with RRMM who have received one to three prior lines of therapy [214]. **DARZALEX FASPRO** is a new subcutaneous formulation of **daratumumab** (**DARZALEX**) which is co-formulated with recombinant human **hyaluronidase**. The drug is administered in significantly less time (i.e., three to five minutes) compared to the hours of intravenous infusion required for **DARZALEX**. **Hyaluronidase** increases the permeability of the subcutaneous tissue. In the doses administered, **hyaluronidase** in **DARZALEX FASPRO** acts locally, and the effects of its action are reversible. The drug was approved as a monotherapy and in combination with four chemotherapy regimens across multiple myeloma, including newly diagnosed, transplant-ineligible patients as well as relapsed or refractory patients [215].

#### 3.1.4. Anti-SLAMF7 Monoclonal Antibody

Signaling lymphocytic activation molecule family member 7 (SLAMF7, also known as CS1, CD319, and CRACC) is a transmembrane receptor that is expressed on hematopoietic cells such as natural killer (NK) cells, B cells, T cells, NK-T cells, dendritic cells, and monocytes. The receptor modulates the function of immune cells through immune-receptor tyrosine-based switch motifs, which mediates interaction with Ewing’s sarcoma-associated transcript 2 (EAT-2). SLAMF7 has both activating and inhibitory functions in NK cells, induces proliferation and the production of autocrine cytokines in B cells, and inhibits the production of proinflammatory cytokines by activated monocytes. Since high-level expression of SLAMF7 is retained in multiple myeloma (MM) cells, implicated in the uncontrolled proliferation of MM cells, the receptor is a promising target for immunotherapy [216]. **Elotuzumab** is a humanized IgG1 monoclonal antibody that binds to SLAMF7 on MM cells and natural killer (NK) cells. **Elotuzumab** exerts antimyeloma effects via direct NK cell activation and NK-mediated antibody-dependent cellular cytotoxicity (ADCC). The drug also prevents the adhesion of MM cells to bone marrow stromal cells, which may disrupt the growth and survival of MM cells [217]. **Elotuzumab** has no significant clinical efficacy as a single agent; however, it is effective in combination with **lenalidomide** and **dexamethasone** or **pomalidomide** and **dexamethasone**, and, thus, received approval in combination with these two chemotherapy regimens for the treatment of adult patients with relapsed or refractory multiple myeloma (RRMM) who have received prior therapies [218].

#### 3.1.5. Anti-PD-1 Monoclonal Antibodies

Programmed death-1 (PD-1) is a cell surface protein belonging to the CD28 family, expressed on activated T cells, B cells, natural killer (NK) cells, and monocytes. Interaction between PD-1 and its ligands PD ligand-1 (PD-L1) and PD ligand-2 (PD-L2) plays an important role in the activation of inhibitory kinases involved in T-cell proliferation, adhesion, or cytokine production and secretion. This interaction causes the suppression of T cells’ response upon antigen exposure, which allows cancer cells to evade immune surveillance. PD-1 ligands are overexpressed in hematologic malignancies, such as multiple myeloma, lymphoma, and various leukemia types, which results in the inhibition of T-cell–mediated immune response in these diseases. Blocking the PD-1/PD-L1 signaling pathway with monoclonal antibodies (mAbs) has an important role in cancer treatment because it restores the efficacy of cancer-specific T cells, allowing them to recognize and counter cancer cells [219]. **Nivolumab** and **pembrolizumab** are the first two FDA-approved mAbs that target PD-1. These drugs selectively impede the interaction between PD-1 and PD-L1, and lead to the release of T cells from pathological immune suppression [220]. **Nivolumab** received accelerated approval for patients with classical Hodgkin lymphoma that has relapsed or progressed after autologous hematopoietic stem cell transplantation (HSCT) and posttransplantation treatment with **brentuximab vedotin**. **Pembrolizumab** was registered as monotherapy to treat pediatric and adult patients with relapsed or refractory classical Hodgkin lymphoma (cHL). Moreover, these drugs have earned a series of other FDA approvals, including for melanoma, non-small cell lung cancer (NSCLC), renal cell carcinoma, head and neck cancer, urothelial carcinoma, hepatocellular carcinoma, esophageal carcinoma, and gastric cancer [221,222].

#### 3.1.6. Anti-CCR4 Monoclonal Antibody

C-C chemokine receptor 4 (CCR4) is a G protein-coupled receptor for small molecular weight chemotactic cytokines, i.e., chemokines, that is expressed on the surface of regulatory T cells (Treg) and type 2 T helper cells (Th2). CCR4 plays an important role in the migration of T cells to the skin during the response to skin inflammation. This receptor is also overexpressed in some subtypes of cutaneous T-cell lymphoma (CTCL), including mycosis fungoides (MF) and Sézary syndrome (SS) [223]. **Mogamulizumab-kpkc** is a first-in-class defucosylated, humanized mAb against CCR4, which is used to treat adult patients with relapsed or refractory MF and SS who received at least one prior systemic therapy. The drug, by selectively binding to CCR4, marks T cells for depletion through antibody-dependent cellular cytotoxicity (ADCC) and destroys target cancer cells [224].

### 3.2. Bispecific Antibody as Anticancer Agent

**Figure 10:**
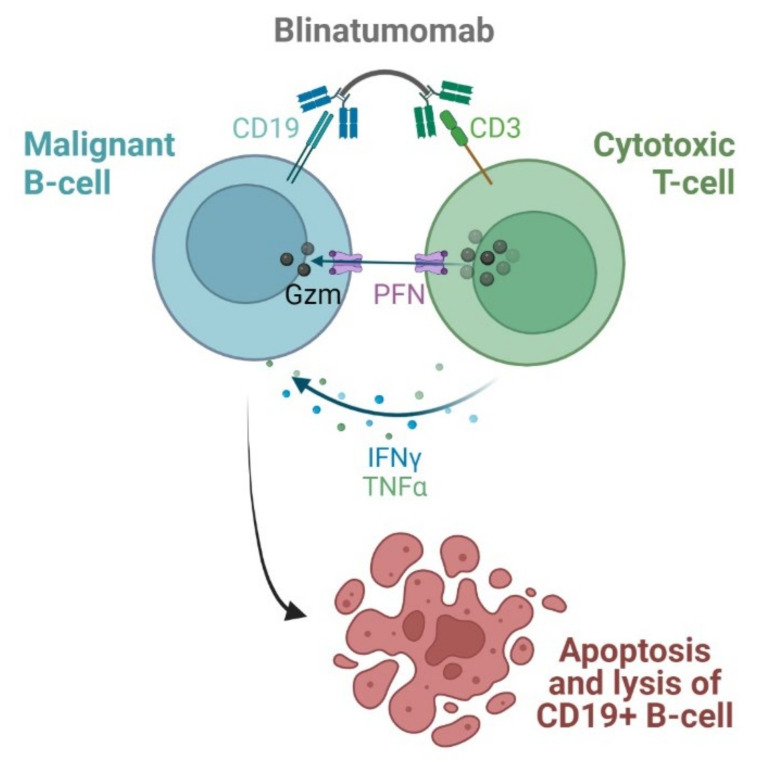
Blinatumomab mechanism of action. **CD19**: cluster of differentiation 19. **CD3**: cluster of differentiation 3. **Gzm**: granzymes. PFN: perforin. **IFNγ**: interferon gamma. **TNFα**: tumor necrosis factor alpha. Created with BioRender.com.

A bispecific antibody (bsAb) is a single, hybrid protein containing two different antibody-based binding specificities, thus limiting complement activation that is responsible for side effects of treatment. The use of bsAb profoundly improves the selectivity and efficacy of therapies of various diseases, including hematological malignancies. Bispecific antibodies differ in structure, size, and method of production [247]. **Blinatumomab** is the first-in-class bispecific T-cell-engager antibody (BiTE) approved to treat children and adults with relapsed or refractory B-cell precursor acute lymphoblastic leukemia (ALL). The drug binds to both CD19 and CD3, which are expressed on the surface of malignant B cells and endogenous T cells, respectively (Figure 10). Subsequently, a cytolytic synapse between B cells and T cells is formed, resulting in the activation of cytotoxic T cells. This induces apoptosis and lysis of the CD19 positive B cells via the release of perforin and granzymes from cytotoxic granules in the T cells. Furthermore, inflammatory cytokines (i.e., interferon γ (IFNγ) and tumor necrosis factor α (TNFα)) are released and T cell proliferation is promoted [248]. The summary of **blinatumomab** characteristics is included in Table 12.

### 3.3. Antibody-Drug Conjugates as Anticancer Agents

**Figure 11:**
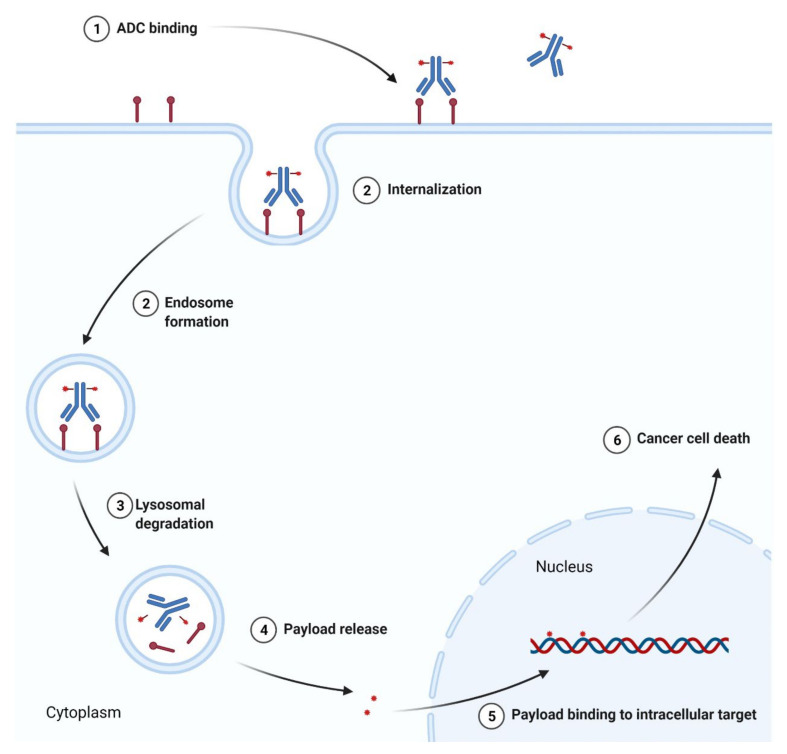
General mechanism of action of antibody-drug conjugates. **ADC**: antibody-drug conjugate. Adapted from “Intracellular layout—endocytosis pathway,” by BioRender.com (2021). Retrieved from https://app.BioRender.com/biorender-templates (accessed on: 8 December 2021).

Antibody-drug conjugate (ADC) is an immunoconjugate consisting of an antibody, a highly potent cytotoxic agent (known as the payload), and a chemical linker. The antibody moiety enables selective delivery of the payload to the target cancer cells by directly binding to specified cancer-associated antigen. Upon the internalization of the ADC-antigen complex into the cancer cell, ADC degradation occurs in the lysosome by the cleavage of the linker, which permits the efficient release of the payload. Finally, the released payload induces target cell death (Figure 11). ADCs confer to reduce off-target toxicity and increase the efficacy of the payloads in patients by limiting their exposure to normal tissues [251]. By the end of 2020, four ADCs had received FDA approval. Among them were antibody-drug conjugate targeting cluster of differentiation 22 (CD22), cluster of differentiation 30 (CD30), cluster of differentiation 79b (CD79b), and B-cell maturation antigen (BCMA), which are summarized in Table 13.

#### 3.3.1. Anti-CD22 Antibody-Drug Conjugate

A cluster of differentiation 22 (CD22) is a transmembrane sialoglycoprotein belonging to the immunoglobulin superfamily. CD22 expressed on mature B cells in a large variety of B-cell malignancies is involved in B cell activation through both a positive and negative regulation of B cell antigen receptor (BCR) signaling as well as in B cell migration and survival [252]. The expression of CD22 antigens is lineage-restricted, is generally not lost during neoplastic transformation, and is absent on normal hematopoietic stem cells, making them ideal targets for therapy of hematologic malignancies. **Inotuzumab ozogamicin** is a drug composed of a semisynthetic derivative of calecheamicin, a toxic product isolated from *Micromonospora echinospora* subsp. *calichensis*, and monoclonal IgG4 antibody targeting CD22. This antibody and calicheamicin derivative, i.e., *N*-acetyl-γ-calicheamicin dimethyl hydrazide (DMH), are covalently connected via a bifunctional acetophenone-butanoic acid linker. The linker forms an amide bond with lysine residues of the monoclonal antibody and a hydrazone linkage with *N*-acetyl-γ-calicheamicin DMH. The hydrolysis of the hydrazone functional group allows releasing of the cytotoxic component into the acidic environment of the lysosomes in cancer cells. Calecheamicin derivative is then activated by the reduction of a disulfide bond in the presence of intracellular glutathione (GSH), a cytoplasmic thiol cofactor. Calecheamicin shows a different mechanism of action from other tubulin-binding classes of cytotoxic agents. The enediyne moiety of calicheamicin, once reduced by cellular thiols, undergoes rearrangement, forming 1,4-benzenoid diradicals. Next, the diradical intermediates cause the abstraction of hydrogen atoms from the phosphodiester bonds of double-stranded DNA and their breaking. Subsequently, this leads to cell cycle arrest between the gap 2 phase and mitosis (G2/M) and apoptotic cell death. Overall, calecheamicin acts by binding to the minor DNA groove and causing double-strand DNA breaks in a manner independent of cell cycle progression, which induces apoptosis even in rapidly dividing cells [253,254]. **Inotuzumab ozogamicin** has been an approved drug since 2017 for the treatment of adults with relapsed or refractory B-cell precursor ALL [255]. 

#### 3.3.2. Anti-CD30 and Anti-CD79b Antibody-Drug Conjugates

A cluster of differentiation 30 (CD30) is a transmembrane glycoprotein, a member of the tumor necrosis factor receptor (TNFR) superfamily, that is expressed on B or T lymphocytes in classical Hodgkin’s lymphoma (cHL) and anaplastic large cell lymphoma (ALCL). CD30 induces pleiotropic biological effects on cell growth and survival through activation of the NF-κB pathway [256]. A cluster of differentiation 79b (CD79b) is a component of the B cell receptor (BCR) complex that is expressed only on mature B cells and B cell malignancies. CD79b plays a key role in downstream BCR signaling [257]. **Brentuximab vedotin** and **polatuzumab vedotin** are drugs consisting of the monoclonal antibody directed against CD30 and CD79b, respectively, and monomethyl auristatin E (MMAE), a potent antimitotic agent, conjugated by a protease-cleavable maleimidocaproyl-valine-citrulline-p-aminobenzyloxycarbonyl linker. After internalization of these antibody-drug conjugates into a cancer cell, the linker is selectively cleaved by lysosomal enzymes and MMAE is released. The antimitotic agent then binds to tubulin and blocks their polymerization, leading to G2/M phase cell cycle arrest and cellular apoptosis [258,259]. Additionally, both drugs induce cancer cells’ death via antibody-mediated cellular phagocytosis (ADCP). **Brentuximab vedotin** is currently used as therapy for patients with systematic ALCL and cHL after the failure of prior combination chemotherapy regimens. The drug is also approved to treat adult patients with primary cutaneous anaplastic large cell lymphoma (pcALCL) and mycosis fungoides (MF) after the failure of prior systemic therapy. Moreover, the drug, in combination with chemotherapy, is the first-line treatment for adult patients with stage III or IV cHL who were previously untreated [260]. Clinical trials indicate that **brentuximab vedotin** followed by **bendamustine** supercharge is a promising treatment option for high-risk Hodgkin lymphoma. [261]. **Polatuzumab vedotin** is used in combination with **bendamustine** plus **rituximab** to treat adults with relapsed or refractory diffuse large B-cell lymphoma (DLBCL) who have previously attempted two other therapies [262].

#### 3.3.3. Anti-BCMA Antibody-Drug Conjugate

B cell maturation antigen (BCMA) is a transmembrane glycoprotein belonging to the tumor necrosis factor receptor superfamily 17 (TNFRSF17). The receptor is overexpressed on multiple myeloma (MM) cells as well as normal plasma cells, but not on other normal tissues. BCMA plays an important role in bone marrow plasma cell survival [263]. **Belantamab mafodotin-blmf** is a first-in-class anti-BCMA ADC indicated for the treatment of MM. The drug comprises an afucosylated IgG1 monoclonal antibody covalently conjugated to the cytotoxic microtubule inhibitor, i.e., monomethyl auristatin F (MMAF), through a maleimidocaproyl linker (mc). The antibody moiety delivers the MMAF to the BCMA-expressing cells. After the internalization of ADC and the release of MMAF via proteolytic degradation, the cytotoxic agent disrupts the formation of microtubules by inhibiting tubulin polymerization. Subsequently, this causes cell cycle arrest in the G2/M phase and apoptosis. Furthermore, **belantamab mafodotin-blmf** induces cancer cell lysis via antibody-dependent cellular cytotoxicity (ADCC) and phagocytosis (ADCP). The drug received FDA approval as a monotherapy for adults with relapsed or refractory MM after at least four prior treatments [264].

### 3.4. Recombinant Immunotoxin as Anticancer Agent

**Figure 12:**
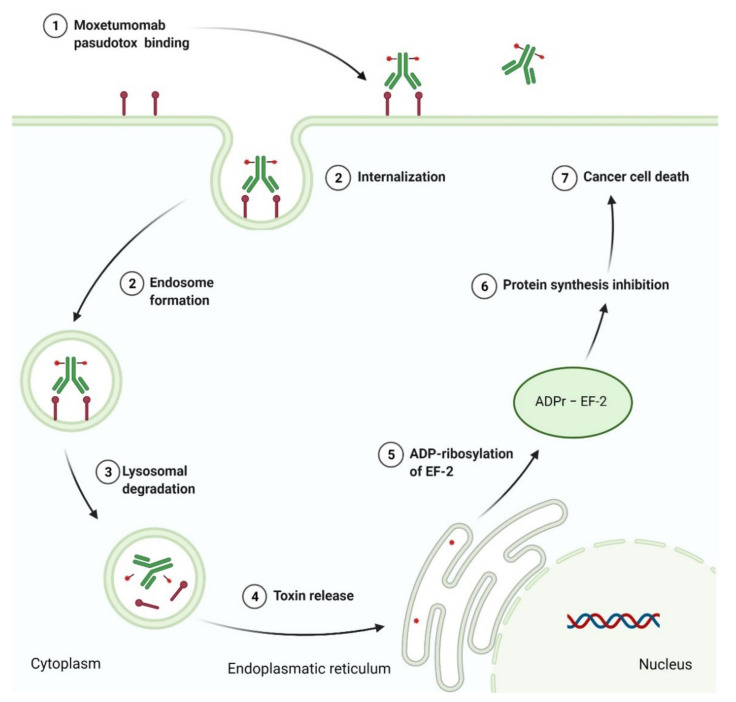
Intoxication of cell by moxetumomab pasudotox. **ADP**: adenosine diphosphate. **ADPr**: adenosine diphosphate ribose. **EF-2**: elongation factor-2. Figure based on the following reference [275]. Adapted from “Intracellular layout—endocytosis pathway,” by BioRender.com (2021). Retrieved from https://app.BioRender.com/biorender-templates (accessed on: 8 December 2021).

Recombinant immunotoxins (RITs) are fusion molecules that contain the Fv fragment of a monoclonal antibody (mAb) connected to the specified protein toxin. The toxin, produced by bacteria, fungi, or plants, is an extremely cytotoxic molecule that rapidly induces apoptotic cell death via inhibition of protein synthesis within the cell cytosol. Immunotoxins demonstrate potent clinical efficacy against various types of cancers, including hematological malignancies [273]. **Moxetumomab pasudotox** is the FDA-approved drug used to treat adult patients with relapsed or refractory hairy cell leukemia (HCL) after at least two prior systemic therapies [274]. **Moxetumomab pasudotox** is composed of the Fv fragment of an anti-CD22 monoclonal antibody genetically fused to PE38, which is a fragment of *Pseudomonas* exotoxin A. The Fv portion of this drug binds to cluster of differentiation 22 (CD22), a transmembrane sialoglycoprotein expressed on a variety of malignant B-cells, and thus delivers toxin moiety directly to target cells. After binding, the recombinant immunotoxin-CD22 complex is internalized into the cell, where the furin catalyzed cleavage of a fragment of PE38 occurs. The released PE38 then catalyzes the adenosine diphosphate (ADP)-ribosylation of the diphthamide residue in elongation factor-2 (EF-2), resulting in a decrease in protein myeloid cell leukemia 1 (MCL1) levels, and thereby cellular apoptosis (Figure 12) [275]. The overall features of the drug are summarized in Table 14.

### 3.5. Enzymes as Anticancer Agents

**Figure 13:**
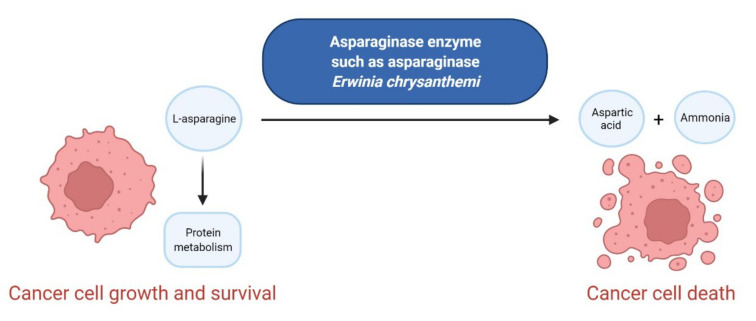
Schematic representation of mechanism of action for asparaginases such as asparaginase *Erwinia chrysanthemi* and calaspargase pegol-mknl. Created with BioRender.com.

Enzymes used as anticancer therapy target amino acid metabolism. They are involved in the degrading of amino acids such as asparagine, arginine, and methionine, for which certain cancerous cells become auxotrophic. The amino acid deprivation leads to the inhibition and impairment of cancer growth [278]. The enzymes approved during the last decade for treatment of hematological malignancies are summarized in Table 15. **Asparaginase *Erwinia chrysanthemi*** is a novel formulation of L-asparaginase, which is the enzyme responsible for the metabolism of L-asparagine derived from the Gram-negative bacteria *Erwinia chrysanthemi*. The drug is immunologically distinct from *Escherichia coli*-derived L-asparaginase and demonstrates higher enzymatic activity. It was developed to avoid toxicities such as hypersensitivity, pancreatitis, and hyperglycemia associated with previously approved L-asparaginase therapies [279]. **Calaspargase pegol-mknl** is also an L-asparagine specific enzyme, but is connected with 31–39 molecules of monomethoxy-polyethylene glycol (mPEG) by a succinimidyl carbonate (SC) linker. This new drug is a long-acting product, which provides for less frequent dosing compared to other L-asparaginase therapies [280]. **Asparaginase *Erwinia chrysanthemi*** received approval to treat patients with acute lymphoblastic leukemia (ALL) who had to discontinue treatment with *Escherichia coli*-derived asparaginase because of hypersensitivity, while **calaspargase pegol-mknl** was approved to treat pediatric and young adult patients with ALL. Both drugs are used as components of a multi-agent chemotherapeutic regimen [281,282]. The mechanism of their action includes hydrolysis of L-asparagine to aspartic acid and ammonia resulting in depleting circulating levels of L-asparagine, which is essential for the protein metabolism of leukemic cells, and thereby their growth and survival (Figure 13). Subsequently, L-asparagine deprivation in leukemic cells induces cell death [282,283].

### 3.6. Chimeric Antigen Receptor T (CAR-T) Cells as Anticancer Agents

**Figure 14:**
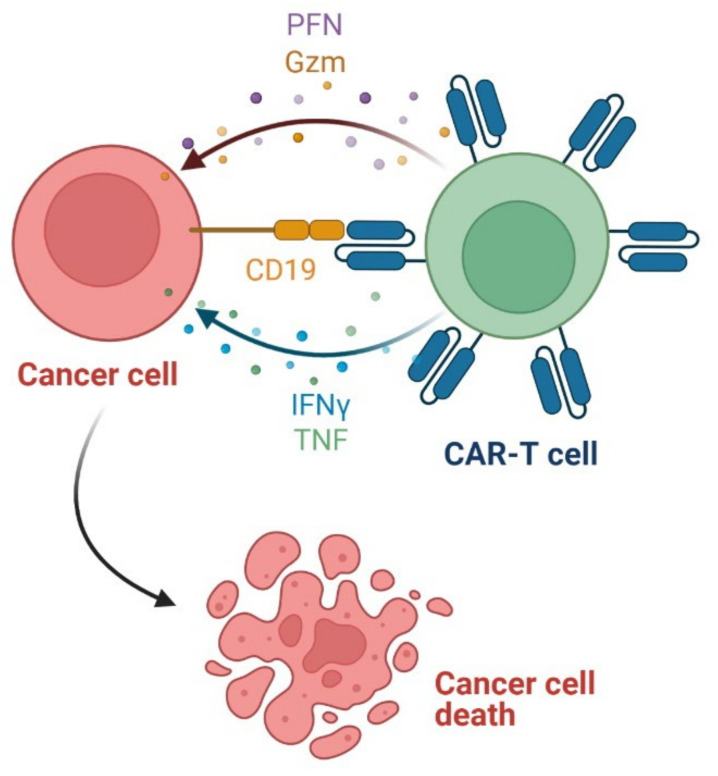
The immune-mediated cytotoxic mechanism of CAR-T cell. **PFN**: perforin. **Gzm**: granzymes. **CD19**: cluster of differentiation 19. **IFNγ**: interferon gamma. **TNF**: tumor necrosis factor. **CAR-T**: chimeric antigen receptor T. Created with BioRender.com.

Chimeric antigen receptor T (CAR-T) cell therapy is a novel, promising immunotherapy treatment of various hematological malignancies. In the CAR-T cell, T lymphocytes separated from the patient’s peripheral blood are engineered with genetic recombinant receptors known as chimeric antigen receptors (CAR). A CAR is a synthetic protein that consists of three parts, i.e., an extracellular antibody-derived domain, a transmembrane domain, and an intracellular T-cell signaling domain. The extracellular domain targets specific tumor-associated antigens (TAAs) while the intracellular domain enhances T-cell proliferation and differentiation. There are four generations of CAR proteins that differ in the structure of the intracellular signaling domain. CAR-T cells lead to cancer cell death mainly via the secretion of cytotoxic granules containing granzymes and perforin, and the release of cytokines (i.e., IFNγ and tumor necrosis factor (TNF)) (Figure 14). CAR-T cells provide antigen recognition in a major histocompatibility complex (MHC)-independent manner. For this reason, the immune response during CAR-T therapy may be out of control and cause some toxic side effects [286,287]. CAR-T therapies which are FDA-approved until 2021, i.e., **tisagenlecleucel**, **axicabtagene ciloleucel**, and **brexucabtagene autoleucel**, are second-generation CD19-directed CAR-T cells (Table 16). **Tisagenlecleucel** is the first genetically modified therapy, which is autologous T-cells modified through lentiviral transduction to express a CAR composed of a murine single-chain antibody fragment (scFv), a transmembrane domain, and an intracellular signaling domain (CD3ζ) fused to a costimulatory domain (4-1BB) [288]. It acts by binding to CD19-expressing cells, then initiating T-cell activation via the CD3ζ component. The 4-1BB domain provides enhancing of the expansion and persistence of **tisagenlecleucel**. This drug is now registered for two distinct indications in relapsed or refractory B-cell acute lymphoblastic leukemia (ALL) and certain types of relapsed or refractory large B-cell lymphomas, including diffuse large B-cell lymphoma (DLBCL), high-grade B-cell lymphoma, and DLBCL arising from follicular lymphoma, for which other therapies have failed [289]. **Axicabtagene ciloleucel** and **brexucabtagene autoleucel** are autologous T-cells that express a CAR, which contains a murine single-chain variable fragment (scFv), a transmembrane domain, and an intracellular signaling domain (CD3ζ) integrated with a costimulatory domain (CD28). The primary difference between these drugs is in their manufacturing processes. With **brexucabtagene autoleucel** specifically, there is an additional T-cell enrichment phase during production, which causes the removal of circulating CD19-expressing cancer cells in the leukapheresis material. This stage prevents potential manufacturing failure in the case of patients with mantle cell lymphoma (MCL), who may have a high number of cancer cells in their peripheral blood and relatively fewer T-cells [290,291]. Hence, **axicabtagene ciloleucel** and **brexucabtagene autoleucel** represent an effective treatment modality in adult patients with various large B-cell lymphomas, such as DLBCL, and relapsed or refractory MCL, respectively. These drugs are infused into the patient after conditioning with lymphodepleting chemotherapy. Their mechanism of action includes binding to CD19-expressing cancer cells, and subsequent activation of downstream signaling pathways through the CD28 and CD3ζ domains, leading to T-cell activation and proliferation, as well as the secretion of inflammatory cytokines and chemokines. This results in the effective eradication of cancer cells [292,293]. While **tisagenlecleucel**, **axicabtagene ciloleucel,** and **brexucabtagene autoleucel** are potent anticancer agents, they may cause severe adverse reactions, including cytokine release syndrome and neurologic toxicities. For this reason, CAR-T therapies are available only through restricted programs under a Risk Evaluation and Mitigation Strategy (REMS), i.e., the KYMRIAH REMS Program and the YESCARTA and TECARTUS REMS Programs [289,292,293].

## 4. Conclusions

Fifty two new drug registrations approved by the FDA were made in the period between 2011 and 2021 for use in therapies of hematological malignancies; 29 of them were for small molecule drugs and 23 of them were for macromolecules. Forty out of all these drugs (21 small molecule and 19 macromolecular drugs) were also approved by the EMA (European Medicines Agency). Depending on the mechanism of action, these drugs can be classified as chemotherapy agents, whose activity is due to disruption of the mitotic and/or DNA replication pathways, and targeted agents, which inhibit the molecular targets involved in cancer cell growth and spread. In the studied period, the number of FDA-approved chemotherapy drugs is significantly smaller (6 drugs) than ones containing targeted molecules (46 drugs) as defined above. The route of administration for these drugs is mainly intravenous (28 drugs) or oral (23 drugs), and only one (1) is subcutaneous injection, which may be administered at home by the patient or a caregiver. Six (6) of these registered drugs (i.e., **ERWINAZE**, **PURIXAN**, **MARQIBO**, **INQOVI**, **ONUREG**, and **VYXEOS**) are medications that contain anticancer active compounds (i.e., **asparaginase *Erwinia chrysanthemi***, **mercaptopurine**, **vincristine sulfate**, **decitabine, azacitidine**, and a fixed-dose combination of **cytarabine** and **daunorubicin**, respectively) and that have been used for more than 10 years; however, their present forms have significantly better pharmacological parameters. In the case of **MARQIBO** and **VYXEOS**, which are liposome-encapsulated chemotherapy agents, delivery efficiency, biocompatibility, and pharmacokinetics are improved. In addition, **VYXEOS** consists of two active compounds in the optimal molar ratio, of which simultaneous delivery to cancer cells improves drug pharmacologic action and reduces the occurrence of resistance. **ONUREG** and **INQOVI** are formulated as film-coated tablets and fixed-dose combination with **cedazuridine**, respectively, that enable their oral administration due to avoiding degradation by cytidine deaminase. A new oral formulation of these drugs induces facilitated dosing, reduces administration side effects, and potentially increases efficacy. In contrast, **PURIXAN** is developed as an oral suspension to the accuracy of drug dose for individual patients. For **ERWINAZE**, there is increased enzymatic activity and reduced adverse events of asparaginase therapy.

All of the 29 presented new small molecule drugs are based on aromatic organic compounds, which, apart from two (2) drugs (i.e., **ixazomib citrate** (**NINLARO**) and **belinostat** (**BELEODAQ**)), contain a heterocyclic system in their structure. Among them, there is a large number of compounds bearing a nitrogen heterocycle ring (28 drugs) and a much smaller number with an oxygen heterocycle ring (6 drugs). A deeper analysis of pharmaceuticals containing nitrogen heterocycles indicates that bicyclic compounds, such as indole, purines, and pyrrolopyrimidines, and six-membered mono ring heterocycles, including pyridine, pyrimidine, pyrazine, and triazine, are the most common in structures of small molecule drugs. Only three (3) medicaments, i.e., **omacetaxine mepesuccinate** (**SYNRIBO**), **vincristine** (**MARQIBO**), and **midostaurin** (**RYDAPT**), are heteropolicyclic compounds. These belong to the class of compounds known as cephalotaxus alkaloids, vinca alkaloids, and indolocarbazoles, respectively. Moreover, almost half (14 drugs) of the drugs approved in the last decade contain chiral active compounds. It is interesting to note that only 1 (1) drug consists of a boron-containing molecule, 5 (5 drugs) are sulfur-containing compounds (i.e., sulfate, sulfonamide, or thioketone), and 10 (10 drugs) contain one or more halogen atoms (specifically fluorine or chlorine) in the structure. The boronic acids group, which is present in a peptidomimetic drug—**ixazomib**, acts as a reversible covalent inhibitor of nucleophilic threonine residues in the active sites of the 20S unit of the 26S proteasome. Subsequently, it blocks the ubiquitin-mediated protein degradation pathway leading to cancer cell death [300]. Other scaffolds found in drugs approved in the studied period are amide bonds and hydroxamic acid moiety. Due to the crucial role of the amide group for the function of many biomolecules, it is often represented in the pharmacophore of biologically active compounds [301]. The authors have noticed that 18 registered small molecules contain the amide functional group. The hydroxamic acid group is found in two (2) drugs (**belinostat** and **panobinostat**) which are HDACs inhibitors. The coordination of the hydroxamic acid to zinc ion in the active site of HDACs is essential to the inhibitory effect of these drugs [302]. Despite the toxicity of the nitrile (CN) and nitro (NO_2_) compounds [303,304], four (4) of the small molecule inhibitors are nitrile-containing pharmaceuticals, and one (1) contains the nitroaromatic compound. These drugs induce anticancer effects by diverse mechanisms including Src and Abl kinases inhibition (**bosutinib**), JAK1 and JAK2 inhibition (**ruxolitinib**), IDH1 inhibition (**ivosidenib**), SMO receptor antagonism (**glasdegib**), and BCL-2 inhibition (**venetoclax**). Since CN and NO_2_ groups have an electron-withdrawing nature, their role is a creation of electron-deficient sites in drug structures and nonspecific dipole interaction with biological nucleophiles present in living organisms, e.g., amino acids, nucleic acids, and enzymes [305,306]. It is worth noting that eight (8) of the small molecule drugs are characterized by a completely new mechanism of action, such as BTK inhibition (**ibrutinib**), PI3K-δ inhibition (**idelalisib**), PI3K- δ/γ inhibition (**dulvalisib**), IDH2 inhibition (**enasidenib**), IDH1 inhibition (**ivosidenib**), EZH2 inhibition (**tazemetostat**), BCL-2 inhibition (**venetoclax**), and XPO1 inhibition (**selinexor**). Furthermore, two (2) drugs contain a fixed-dose combination of two anticancer active ingredients. **VYXEOS** consists of topoisomerase II inhibitor—**daunorubicin**, and pyrimidine base antagonist—**cytarabine**. **INQOVI** contains **decitabine**, which inhibits DNA methyltransferases, and **cedazuridine**, which protects decitabine from being broken down by cytidine deaminase.

At the same time, it should be emphasized that more and more drugs from the macromolecular group (such as antibodies, enzymes, and CAR-T cells) are starting to be applied in anticancer therapy and it is supposed that this tendency will continue to the advantage of this composite biomolecular group of drugs. Among the 23 FDA-approved macromolecule drugs, more than half (12 drugs) are monoclonal antibodies (mAbs). Only one (1 drug) monoclonal antibody, i.e., **mogamulizumab-kpkc**, is referred to as a first-in-class drug, characterized by a novel anti-CCR4 mechanism of action. In contrast, three (3) mAbs (i.e., **TRUXIMA**, **RUXIENCE**, and **RIABNI**) are biosimilars of **rituximab** (**RITUXAN**), which is the first monoclonal antibody used in clinics for the treatment of hematological malignancies. Biosimilars of **rituximab** provide more affordable monoclonal antibody therapy and increase access for patients to receive optimal care. To improve the therapeutic efficacy of mAbs, various modifications have been developed, including currently approved immunoconjugates (five (5) antibody-drug conjugates and one (1) recombinant immunotoxin) and one (1) bispecific monoclonal antibody. There are three main structural elements of immunoconjugates: antibody directed against different types of cancer cell antigens; linker and payload, which is either cytotoxic agent, i.e., microtubule inhibitor (monomethyl auristatin F), antimitotic agent (monomethyl auristatin E), or DNA-damaging antibiotic derivative (*N*-acetyl-γ-calicheamicin dimethyl hydrazide); toxin (*Pseudomonas* exotoxin A). The analysis of linker structures indicates that they consist of the following chemical motifs: acid cleavable hydrazone, reducible disulfide, enzyme cleavable dipeptide with spacer unit (p-aminobenzyloxycarbonyl group), and non-cleavable maleimidocaproyl group. It is worth noting that the FDA registered, for the first time, the bispecific T-cell-engager (BiTE) antibody **blinatumomab** in 2014, and, in 2020, the first anti-BCMA antibody-drug conjugate—**belantamab mafodotin-blmf**. In the last decade, the FDA also approved the first-ever genetically modified CAR-T therapy. By the end of 2020, three (3) CAT-T therapies (i.e., **KYMRIAH**, **YESCARTA**, and **TECARTUS**) were used to treat certain types of leukemia and lymphoma. CAR-T cells differ significantly from other anticancer drugs because of their ability to proliferate and expand. They can persist for months or even years in a patient’s blood and, for this reason, are called “living drugs” [288]. CAR-T cells are the most promising type of anticancer therapy, especially for patients with relapsed or refractory hematological malignancies who do not respond to standard treatments.

## Figures and Tables

**Table 1:** Features of the tyrosine kinase (TK) inhibitors approved by the Food and Drug Administration (FDA) from 2011 to 2021. The order of drugs is tabulated in order of most recent to oldest registration date.

No.	Generic Name of Drug	Brand Name and Company	First FDA/EMA Approved Date	Structure	Molecular Target	Route of Administration	Indication	Adverse Effects	Reference
1	Zanubrutinib	BRUKINSA BeiGene, Ltd., Beijing, China	FDA: 14 November 2019 EMA: 29 May 2019	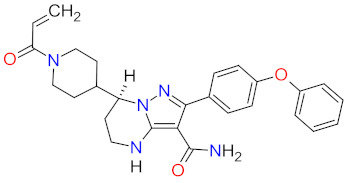	BTK ^1^	Oral	Mantle Cell Lymphoma	Decreased neutrophil count, anemia, neutropenia, pneumonia, decreased platelet count, upper respiratory tract infection, rash, bruising, diarrhea, cough	[39,40]
2	Acalabrutinib	CALQUENCE AstraZeneca, Cambridge, UK	FDA: 31 October 2017 EMA: 5 November 2020	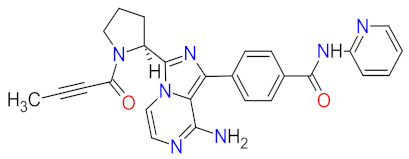	BTK ^1^	Oral	Mantle Cell Lymphoma, Chronic Lymphocytic Leukemia, Small Lymphocytic Lymphoma	Headache, diarrhea, fatigue, nausea, contusion, neutropenia, anemia, pneumonia, thrombocytopenia	[31,41,42]
3	Ibrutinib	IMBRUVICA AbbVie Inc., Lake Bluff, IL, USA	FDA: 13 November 2013 EMA: 21 October 2014	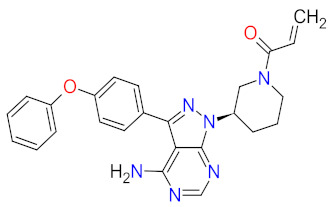	BTK ^1^	Oral	Mantle Cell Lymphoma, Chronic Lymphocytic Leukemia, Waldenström’s Macroglobulinemia, Small Lymphocytic Lymphoma, Marginal Zone Lymphoma	Diarrhea, fatigue, nausea, dyspnea, constipation, peripheral edema, upper respiratory tract infection, rash, cough, arthralgia, vomiting, decreased appetite, thrombocytopenia, neutropenia, anemia, pneumonia, dehydratation	[30,43,44]
4	Bosutinib	BOSULIF Pfizer Inc., New York, NY, USA	FDA: 4 September 2012 EMA: 27 March 2013	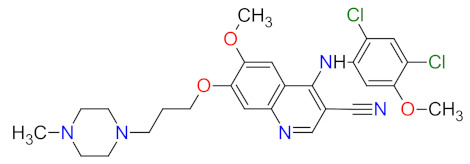	Src ^2^, Abl ^3^	Oral	Chronic Myelogenous Leukemia	Diarrhea, nausea, abdominal pain, vomiting, thrombocytopenia, anemia, neutropenia	[45,46,47]

^1^ **BTK**: Bruton’s tyrosine kinase. ^2^ **Src**: non-receptor Sarcoma kinase. ^3^ **Abl**: Abelson kinase.

**Table 2:** Features of the multi kinase inhibitors approved by the Food and Drug Administration (FDA) from 2011 to 2021. The order of drugs is tabulated in order of most recent to the oldest registration date.

No.	Generic Name of Drug	Brand Name and Company	First FDA/EMA Approved Date	Structure	Molecular Target	Route of Administration	Indication	Adverse Effects	Reference
1	Fedratinib	INREBIC Celgene Corporation, Summit, NJ, USA	FDA: 16 August 2019 EMA: 8 February 2021	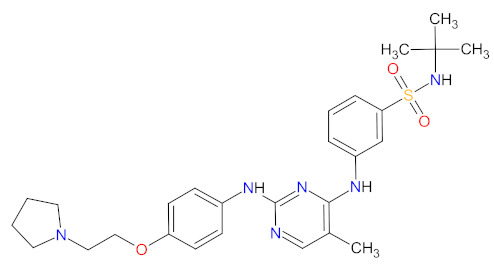	JAK2 ^2^	Oral	Myelofibrosis	Diarrhea, nausea, vomiting, constipation, anemia, thrombocytopenia	[66] ^1^, [67,68]
2	Gilteritinib	XOSPATA Astellas Pharma US, Inc., Northbrook, IL, USA	FDA: 28 November 2018 EMA: 24 October 2019	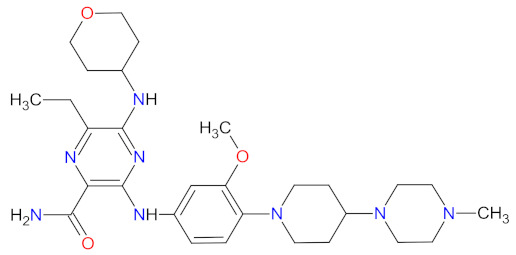	FLT3 ^3^, AXL ^4^, ALK ^5^	Oral	Acute Myeloid Leukemia	Myalgia, arthralgia, increased levels of transaminases, fatigue, malaise, fever, diarrhea, dyspnea, edema, rash, pneumonia, sepsis, renal impairment	[69,70]
3	Midostaurin	RYDAPT Novartis Pharmaceuticals Corporation, East Hanover, NJ, USA	FDA: 28 April 2017 EMA: 18 September 2017	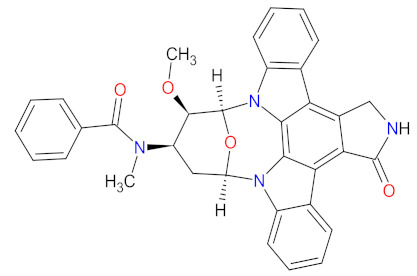	c-Kit ^6^, PDGFRA ^7^, PDGFRB ^8^, FLT3 ^3^, PKC ^9^, CDK1 ^10^, SYK ^11^, VEGFR-2 ^12^	Oral	Acute Myeloid Leukemia, Cutaneous Mastocytosis	Febrile neutropenia, nausea, vomiting, diarrhea, edema, mucositis, headache, device-related infection, abdominal pain, fatigue, pyrexia, dyspnea, musculoskeletal pain, constipation, epistaxis, upper respiratory tract infection, petechial, hyperglycemia,	[71,72]
4	Ponatinib	ICLUSIG Ariad Pharmaceuticals, Inc., Cambridge, MA, USA	FDA: 14 December 2012 EMA: 1 July 2013	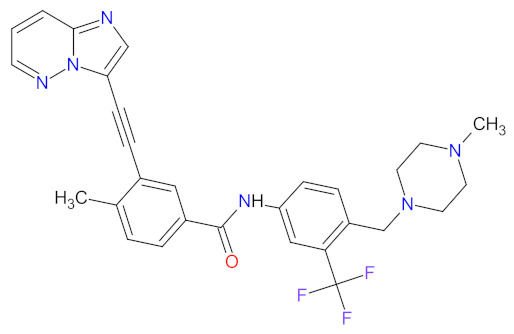	BCR-ABL ^13^, VEGFRs ^14^, FGFRs ^15^, PDGFRs ^16^, RET ^17^, c-Kit ^6^, TIE2 ^18^, FLT3 ^3^	Oral	Chronic Myelogenous Leukemia, Acute Lymphoblastic Leukemia	Hypertension, cardiac failure, abdominal pain, constipation, diarrhea, oral mucositis, febrile neutropenia, fatigue, pneumonia, headache, peripheral neuropathy, dizziness, pleural effusion, cough, dyspnea, rush, dry skin, arthralgia, myalgia, spasms, decreased appetite, edema, weight loss, insomnia	[73,74]
5	Ruxolitinib	JAKAFI Incyte Corporation, Wilmington, DE, USA	FDA: 16 November 2011 EMA: 23 August 2012	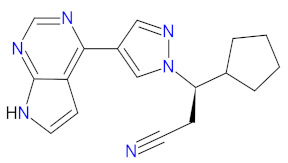	JAK1 ^19^, JAK2 ^2^	Oral	Myelofibrosis, Polycythemia Vera, Graft-versus-host disease	Anemia, thrombocytopenia, neutropenia	[75,76]

^1^ Wrong chemical structure of the drug is given in the reference. ^2^ **JAK2**: Janus kinase 2. ^3^ **FLT3**: FMS-like tyrosine kinase-3. ^4^ **AXL**: AXL receptor tyrosine kinase. ^5^ **ALK**: anaplastic lymphoma kinase. ^6^ **c-Kit**: mast/stem cell growth factor receptor. ^7^ **PDGFRA**: platelet-derived growth factor receptor α. ^8^ **PDGFRB**: platelet-derived growth factor receptor β. ^9^ **PKC**: protein kinase C. ^10^ **CDK1**: cyclin-dependent kinase 1. ^11^ **SYK**: spleen tyrosine kinase. ^12^ **VEGFR-2**: vascular endothelial growth factor receptor-2. ^13^ **BCR-ABL**: BCR-ABL fusion protein. ^14^ **VEGFRs**: vascular endothelial growth factor receptors. ^15^ **FGFRs**: fibroblast growth factor receptors. ^16^ **PDGFRs**: platelet-derived growth factor receptors. ^17^ **RET**: receptor tyrosine kinase rearranged during transfection. ^18^ **TIE2**: tunica interna endothelial cell kinase 2. ^19^ **JAK1**: Janus kinase 1.

**Table 3:** Features of the phosphatidylinositol-3 kinase (PI3K) inhibitors approved by the Food and Drug Administration (FDA) from 2011 to 2021. The order of drugs is tabulated in order of most recent to oldest registration date.

No.	Generic Name of Drug	Brand Name and Company	First FDA/EMA Approved Date	Structure	Molecular Target	Route of Administration	Indication	Adverse Effects	Reference
1	Duvelisib	COPIKTRA Verastem, Inc. Needham, MA, USA	FDA: 24 September 2018 EMA: 19 May 2021	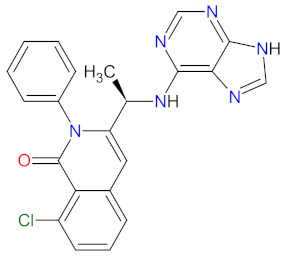	PI3K-δ ^1^, PI3K-γ ^2^	Oral	Chronic Lymphocytic Leukemia, Follicular Lymphoma	Neutropenia, thrombocytopenia, anemia, diarrhea, pyrexia, nausea, vomiting, anorexia	[84,85]
2	Copanlisib	ALIQOPA Bayer HealthCare Pharmaceuticals Inc., HanoverWhippany, NJ, USA	FDA: 14 September 2017 EMA: Not approved	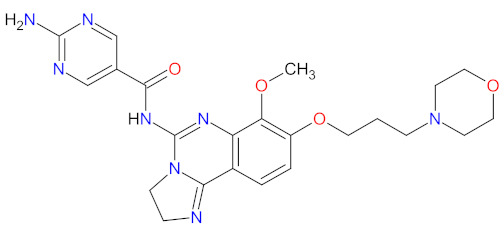	PI3K-α ^3^, PI3K-δ ^1^	Intravenous infusion	Follicular Lymphoma	Hyperglycemia, hypertension, infections, neutropenia	[86,87]
3	Idelalisib	ZYDELIG Gilead Sciences, Inc., Foster City, CA, USA	FDA: 23 July 2014 EMA: 18 September 2014	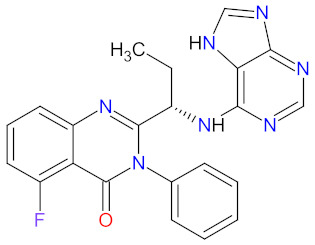	PI3K-δ ^1^	Oral	Chronic Lymphocytic Leukemia, Follicular Lymphoma	Diarrhea, nausea, vomiting, fatigue, headache, pneumonia, chill, dyspnea, rash, neutropenia, pyrexia, sepsis, decreased neutrophil count, hypertriglyceridemia, hyperglycemia, elevated alanine and aspartate transaminases	[88,89]

^1^ **PI3K-δ**: phosphatidylinositol 3-kinase delta. ^2^ **PI3K-γ**: phosphatidylinositol 3-kinase gamma. ^3^ **PI3K-α**: phosphatidylinositol 3-kinase alpha.

**Table 4:** Features of the other enzymes inhibitors approved by the Food and Drug Administration (FDA) from 2011 to 2021. The order of drugs is tabulated in order of most recent to oldest registration date.

No.	Generic Name of Drug	Brand Name and Company	First FDA/EMA Approved Date	Structure	Molecular Target	Route of Administration	Indication	Adverse Effects	Reference
1	Azacitidine	ONUREG Bristol-Myers Squibb Company, New York, NY, USA	FDA: 1 September 2020 EMA: 17 June 2021	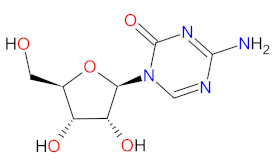	DNMTs ^1^, RNMTs ^2^	Oral	Acute Myeloid Leukemia	Nausea, vomiting, diarrhea, fatigue, constipation, pneumonia, arthralgia, decreased appetite, febrile neutropenia, dizziness, abdominal pain.	[112,114]
2	Tazemetostat	TAZVERIK Epizyme, Inc., Cambridge, MA, USA	FDA: 23 January 2020 EMA: Not approved	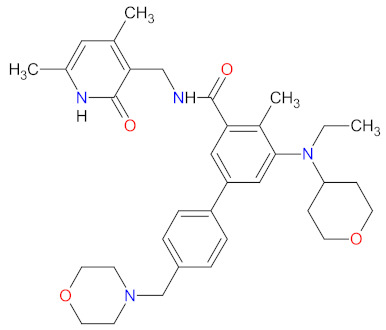	EZH2 ^3^	Oral	Epithelioid Sarcoma, Follicular Lymphoma	Fatigue, nausea, decreased appetite, vomiting, constipation, upper respiratory tract infection, abdominal pain, musculoskeletal pain	[106,115]
3	Ivosidenib	TIBSOVO Agios Pharmaceuticals, Inc., Cambridge, MA, USA	FDA: 20 July 2018 EMA: Not approved	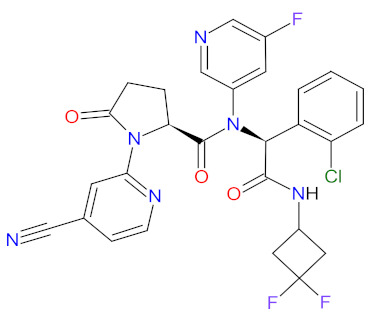	IDH1 ^4^	Oral	Acute Myeloid Leukemia	Diarrhea, leukocytosis, nausea, fatigue, dyspnea, electrocardiogram QT prolonged, edema, anemia, pyrexia, cough, febrile neutropenia, isocitrate dehydrogenase differentiation syndrome	[116,117]
4	Enasidenib	IDHIFA Celgene Corporation, Summit, MA, USA	FDA: 1 August 2017 EMA: Not approved	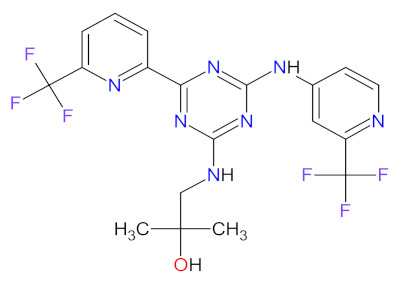	IDH2 ^5^	Oral	Acute Myeloid Leukemia	Elevated bilirubin, nausea, diarrhea, decreased appetite, vomiting	[118,119]
5	Panobinostat	FARYDAK, Novartis Pharmaceuticals Corporation, East Hanover, NJ, USA	FDA: 23 February 2015 EMA: 28 August 2015	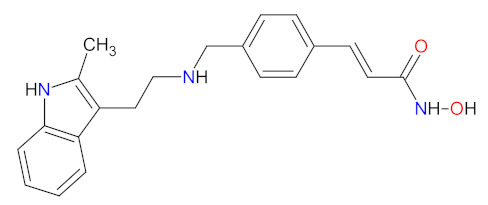	HDAC ^6^	Oral	Multiple Myeloma	Diarrhea, fatigue, nausea, peripheral edema, decreased appetite, pyrexia, vomiting, thrombocytopenia, lymphopenia, leukopenia, neutropenia, anemia, hypophosphatemia, hypokalemia, hyponatremia, increased creatinine	[120,121]
6	Belinostat	BELEODAQ, Spectrum Pharmaceuticals, Inc., Henderson, NV, USA	FDA: 3 July 2014 EMA: Not approved	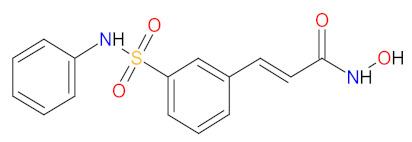	HDAC ^6^	Intravenous infusion	Peripheral T-cell Lymphoma	Nausea, vomiting, diarrhea, fatigue, pyrexia, anemia, constipation, dyspnea, rash, peripheral edema	[122,123]

^1^ **DNMTs**: DNA methyltransferases. ^2^ **RNMTs**: RNA methyltransferases. ^3^ **EZH2**: enhancer of zeste homolog 2. ^4^ **IDH1**: isocitrate dehydrogenase 1. ^5^ **IDH2**: isocitrate dehydrogenase 2. ^6^ **HDAC**: histone deacetylase.

**Table 5:** Features of the various receptor antagonists approved by the Food and Drug Administration (FDA) from 2011 to 2021.

No.	Generic Name of Drug	Brand Name and Company	First FDA/EMA Approved Date	Structure	Molecular Target	Route of Administration	Indication	Adverse Effects	Reference
1	Glasdegib	DAURISMO Pfizer Inc., New York, NY, USA	FDA: 21 November 2018 EMA: 26 June 2020	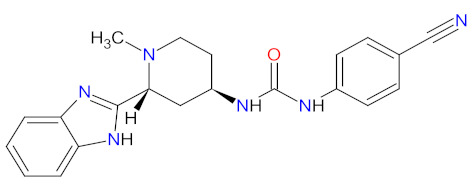	SMO receptor ^1^	Oral	Acute Myeloid Leukemia	Anemia, febrile neutropenia, thrombocytopenia	[129,130]

^1^ **SMO** receptor: smoothened receptor.

**Table 6:** Features of the various proteins inhibitors approved by the Food and Drug Administration (FDA) from 2011 to 2021. The order of drugs is tabulated in order of most recent to oldest registration date.

No.	Generic Name of Drug	Brand Name and Company	First FDA/EMA Approved Date	Structure	Molecular Target	Route of Administration	Indication	Adverse Effects	Reference
1	Selinexor	XPOVIO Karyopharm Therapeutics Inc., Newton Centre, MA, USA	FDA: 3 July 2019 EMA: 26 March 2021	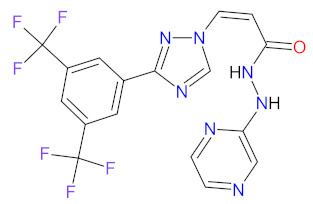	XPO1 ^1^	Oral	Multiple Myeloma, Diffuse Large B-cell Lymphoma	Thrombocytopenia, fatigue, nausea, anemia, decreased appetite, decreased weight, diarrhea, vomiting, hyponatremia, neutropenia, leukopenia, constipation, dyspnea and upper respiratory tract infection	[149,150]
2	Venetoclax	VENCLEXTA AbbVie Inc., Lake Bluff, IL, USA	FDA: 11 April 2016 EMA: 5 December 2016	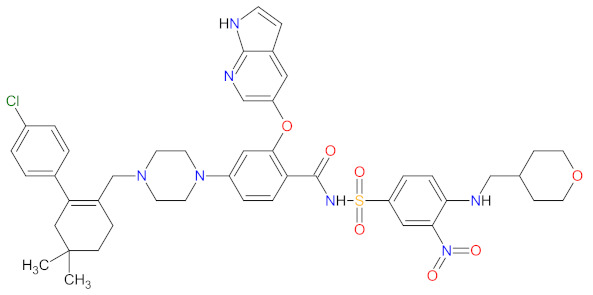	BCL-2 ^2^	Oral	Chronic Lymphocytic Leukemia, Acute Myeloid Leukemia	Neutropenia, diarrhea, nausea, anemia, thrombocytopenia, upper respiratory tract infection, fatigue	[151,152]
3	Ixazomib citrate	NINLARO Takeda Pharmaceuticals U.S.A., Inc., Deerfield, IL, usa	FDA: 20 November 2015 EMA: 21 November 2016	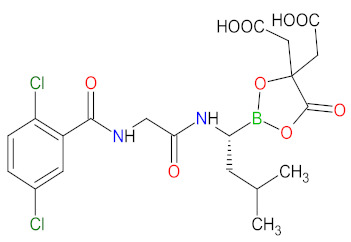	Proteasome	Oral	Multiple Myeloma	Diarrhea, constipation, thrombocytopenia, peripheral neuropathy, nausea, peripheral edema, vomiting, back pain, rash	[153,154]
4	Vincristine sulfate	MARQIBO Talon Therapeutics Inc., Irvine, CA, USA	FDA: 9 August 2012 EMA: Not approved	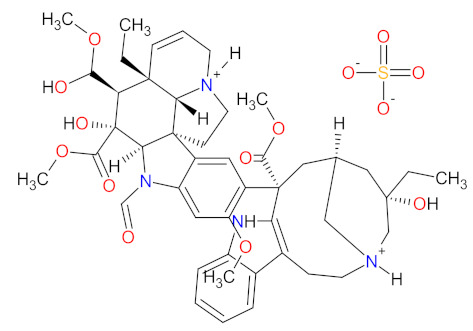	Tubulin	Intravenous	Acute Lymphoblastic Leukemia	Constipation, nausea, pyrexia, fatigue, peripheral neuropathy, febrile neutropenia, diarrhea, anemia, decreased appetite, insomnia	[155,156,157]
5	Carfilzomib	KYPROLIS Amgen Inc., Sauzend Oaks, CA, USA	FDA: 20 July 2012 EMA: 19 November 2015	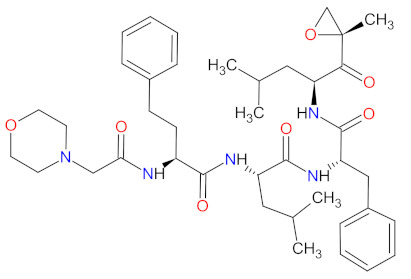	Proteasome	Intravenous	Multiple Myeloma	Fatigue, anemia, nausea, thrombocytopenia, dyspnea, diarrhea, pyrexia	[158,159]

^1^ **XPO1**: exportin-1. ^2^ **BCL-2**: B-cell leukemia/lymphoma-2 proteins.

**Table 7:** Features of the protein translation inhibitor approved by the Food and Drug Administration (FDA) from 2011 to 2021.

No.	Generic Name of Drug	Brand Name and Company	First FDA/EMA Approved Date	Structure	Molecular Target	Route of Administration	Indication	Adverse Effects	Reference
1	Omacetaxine mepesuccinate	SYNRIBO Teva Pharmaceuticals, Tel Aviv, Israel	FDA: 26 October 2012 EMA: Not approved	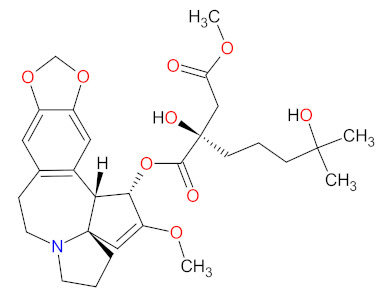	Protein level	Subcutaneous injection	Chronic Myelogenous Leukemia	Thrombocytopenia, anemia, diarrhea, pyrexia, fatigue, nausea, neutropenia, injection site reaction, infection, lymphopenia	[164,165]

**Table 8:** Features of the purine antagonist approved by the Food and Drug Administration (FDA) from 2011 to 2021.

No.	Generic Name of Drug	Brand Name and Company	First FDA/EMA Approved Date	Structure	Molecular Target	Route of Administration	Indication	Adverse Effects	Reference
1	Mercaptopurine	PURIXAN Nova Laboratories, Ltd., Leicester, UK	FDA: 28 April 2014 EMA: 9 March 2012	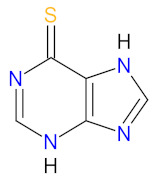	DNA ^1^	Oral	Acute Lymphoblastic Leukemia	Anemia, neutropenia, thrombocytopenia	[170,172,173]

^1^ **DNA**: deoxyribonucleic acid.

**Table 9:** Features of the immunomodulatory drug approved by the Food and Drug Administration (FDA) from 2011 to 2021.

No.	Generic Name of Drug	Brand Name and Company	First FDA/EMA Approved Date	Structure	Molecular Target	Route of Administration	Indication	Adverse Effects	Reference
1	Pomalidomide	POMALYST Bristol-Myers Squibb Company, New York, NY, USA	FDA: 8 February 2013 EMA: 5 August 2013	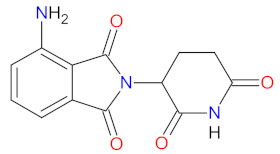	CRBN ^1^	Oral	Multiple Myeloma, Kaposi’s Sarcoma	Neutropenia, thrombocytopenia, anemia, fatigue	[179,180]

^1^ **CRBN**: protein cereblon.

**Table 10:** Features of the dual-drugs approved by the Food and Drug Administration (FDA) from 2011 to 2021. The order of drugs is tabulated in order of most recent to oldest registration date.

No.	Generic Name of Drug	Brand Name and Company	First FDA/EMA Approved Date	Structure	Molecular Target	Route of Administration	Indication	Adverse Effects	Reference
1	Decitabine	INQOVI Astex Pharmaceuticals, Taiho Oncology, and Otsuka Pharmaceutical, Tokyo, Japan	FDA: 7 July 2020 EMA: Not approved	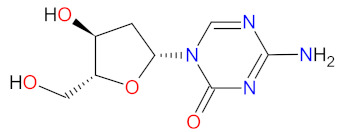	DNMTs ^1^	Oral	Myelodysplastic Syndrome	Fatigue, constipation, hemorrhage, myalgia, nausea, arthralgia, pneumonia, sepsis, decreased leukocytes, decreased platelet count, decreased neutrophil count	[193]
	Cedazuridine			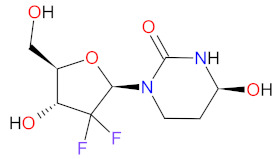	CDA ^2^				
2	Cytarabine	VYXEOS Jazz Pharmaceuticals plc, Dublin, Ireland	FDA: 3 August 2017 EMA: 23 August 2018	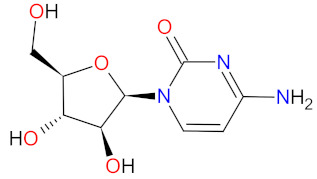	DNA ^3^	Intravenous	Acute Myeloid Leukemia	Hemorrhagic events, febrile neutropenia, rash, edema, nausea, mucositis, diarrhea, constipation, musculoskeletal pain, fatigue, abdominal pain, dyspnea, headache, cough, decreased appetite, arrhythmia, pneumonia, bacteremia, chills, sleep disorders, vomiting	[194,195]
	Daunorubicin			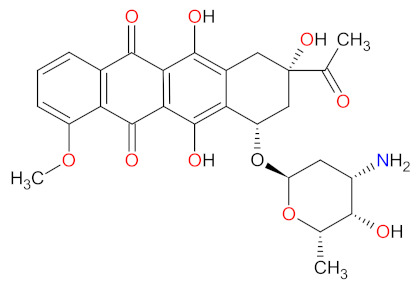	TOP2 ^4^				

^1^ **DNMTs**: DNA methyltransferases. ^2^ **CDA**: cytidine deaminase. ^3^ **DNA**: deoxyribonucleic acid. ^4^ **TOP2**: topoisomerase II.

**Table 11:** Features of the monoclonal antibody drugs approved by the Food and Drug Administration (FDA) from 2011 to 2021. The order of drugs is tabulated in order of most recent to oldest registration date.

No.	Generic Name of Drug	Brand Name and Company	First FDA/EMA Approved Date	Class	Molecular Target	Route of Administration	Indication	Adverse Effects	Reference
1	Rituximab-arrx	RIABNI Amgen Inc., Sauzend Oaks, CA, USA	FDA: 17 December 2020 EMA: Not approved	Chimeric mouse/human immunoglobulin G1 kappa (IgG1 κ) monoclonal antibody	CD20 ^1^	Intravenous	Non-Hodgkin’s Lymphoma, Chronic Lymphocytic Leukemia	Infusion-related reactions, fever, lymphopenia, chills, infection, asthenia, neutropenia	[207]
2	Tafasitamab-cxix	MONJUVI MorphoSys AG, Planegg, Germany	FDA: 31 July 2020 EMA: 26 August 2021	Humanized immunoglobulin G1/2 (IgG1/2) hybrid monoclonal antibody	CD19 ^2^	Intravenous	Diffuse Large B-Cell Lymphoma	Neutropenia, fatigue, anemia, diarrhea, thrombocytopenia, cough, pyrexia, peripheral edema, respiratory tract infection, decreased appetite	[199,225]
3	Daratumumab and hyaluronidase-fihj	DARZALEX FASPRO Janssen Pharmaceuticals, Inc., Belce, Belgium	FDA: 1 May 2020 EMA:Not approved	Humanized immunoglobulin G1 kappa (IgG1 κ) monoclonal antibody and an endoglycosidase	CD38 ^3^	Intravenous	Multiple Myeloma	Upper respiratory tracts infection, constipation, nausea, fatigue, pyrexia, peripheral sensory neuropathy, diarrhea, cough, insomnia, vomiting, back pain, muscle spasms, pneumonia, dyspnea	[215,226]
4	Isatuximab	SARCLISA Sanofi, Paris, France	FDA: 2 March 2020 EMA: 30 May 2020	Chimeric mouse/human immunoglobulin G1 kappa (IgG1 κ) monoclonal antibody	CD38 ^3^	Intravenous	Multiple Myeloma	Infusion reactions, upper respiratory tract infections, bronchitis, pneumonia	[227,228]
5	Rituximab-pvvr	RUXIENCE Pfizer Inc., New York, NY, USA	FDA: 23 July 2019 EMA: 1 April 2020	Chimeric mouse/human immunoglobulin G1 kappa (IgG1 κ) monoclonal antibody	CD20 ^1^	Intravenous	Non-Hodgkin’s Lymphoma, Chronic Lymphocytic Leukemia	Infusion-related reactions, fever, lymphopenia, chills, infection, asthenia, neutropenia	[206,229]
6	Rituximab-abbs	TRUXIMA Celltrion, Inc., Incheon, Korea	FDA: 28 November 2018 EMA: 17 February 2017	Chimeric mouse/human immunoglobulin G1 kappa (IgG1 κ) monoclonal antibody	CD20 ^1^	Intravenous	Non-Hodgkin’s Lymphoma	Infusion-related reactions, fever, lymphopenia, chills, infection, asthenia	[230,231]
7	Mogamulizumab-kpkc	POTELIGEO Kyowa Kirin, Inc., Bedminster, NJ, USA	FDA: 8 August 2018 EMA: 22 November 2018	Humanized immunoglobulin G1 kappa (IgG1 κ) monoclonal antibody	CCR4 ^4^	Intravenous	Mycosis Fungoides, Sézary syndrome	Rash, infusion related reactions, fatigue, diarrhea, musculoskeletal pain, and upper respiratory tract infection	[232,233]
8	Elotuzumab	EMPLICITI Bristol-Myers Squibb Company and AbbVie, Lake Bluff, IL, USA	FDA: 30 November 2015 EMA: 11 May 2016	Humanized immunoglobulin G1 (IgG1) monoclonal antibody	SLAMF7 ^5^	Intravenous	Multiple Myeloma	Fatigue, diarrhea, pyrexia, constipation, cough, peripheral neuropathy, nasopharyngitis, upper respiratory tract infection, decreased appetite, pneumonia	[234,235]
9	Daratumumab	DARZALEX Janssen Biotech, Inc., Horsham, PA, USA	FDA: 16 November 2015 EMA: 20 May 2016	Humanized immunoglobulin G1 kappa (IgG1 κ) monoclonal antibody	CD38 ^3^	Intravenous	Multiple Myeloma	Infusion-related reactions, lymphopenia, neutropenia, thrombocytopenia, anemia	[236,237]
10	Nivolumab	OPDIVO Bristol-Myers Squibb Company, New York, NY, USA	FDA: 22 December 2014 EMA: 19 June 2015	Human immunoglobulin G4 kappa (IgG4 κ) monoclonal antibody	PD-1 ^6^	Intravenous	Hodgkin’s Lymphoma	Fatigue, upper respiratory tract infection, pyrexia, diarrhea, cough	[238,239,240]
11	Pembrolizumab	KEYTRUDA Merck, Kenilworth, NJ, USA	FDA: 4 September 2014 EMA: 17 July 2015	Humanized immunoglobulin G4 (IgG4) monoclonal antibody	PD-1 ^6^	Intravenous	Primary Mediastinal Large B-Cell Lymphoma, Hodgkin’s Lymphoma	Fatigue, cough, pruritus, nausea, rash, decreased appetite, constipation, arthralgia, diarrhea, anemia, hyperglycemia, hyponatremia, hypoalbuminemia, hypertriglyceridemia,, hypocalcaemia, elevated aspartate transaminase	[241,242,243,244]
12	Obinutuzumab	GAZYVA Genentech, Inc., South San Francisco, CA, USA	FDA: 1 November 2013 EMA: 23 July 2014.	Humanized immunoglobulin G1 (IgG1) monoclonal antibody	CD20 ^1^	Intravenous	Chronic Lymphocytic Leukemia, Follicular Lymphoma	Infusion reactions, neutropenia	[245,246]

^1^ **CD20**: cluster of differentiation 20. ^2^ **CD19**: cluster of differentiation 19. ^3^ **CD38**: Cluster of differentiation 38. ^4^ **CCR4**: CC chemokine receptor 4. ^5^ **SLAMF7**: Signaling Lymphocyte Activation Molecule Family member 7. ^6^ **PD-1**: programmed death receptor-1.

**Table 12:** Features of the bispecific monoclonal antibody drug approved by the Food and Drug Administration (FDA) from 2011 to 2021.

No.	Generic Name of Drug	Brand Name and Company	First FDA/EMA Approved Date	Class	Molecular Target	Route of Administration	Indication	Adverse Effects	Reference
1	Blinatumomab	BLINCYTO Amgen Inc., Thousand Oaks, CA, USA	FDA: 3 December 2014 EMA: 23 November 2015	Bispecific T-cell engaging (BiTE) antibody	CD19 ^1^, CD3 ^2^	Intravenous	Acute Lymphoblastic Leukemia	Pyrexia, headache, peripheral edema, nausea, febrile neutropenia, hypokalemia, constipation, febrile neutropenia, pneumonia, device-related infection, tremor, encephalopathy, infection, overdose, confusion, *Staphylococcal* bacteremia, headache	[249,250]

^1^ **CD19**: cluster of differentiation 19. ^2^ **CD3**: cluster of differentiation 3.

**Table 13:** Features of the antibody-drug conjugates approved by the Food and Drug Administration (FDA) from 2011 to 2021. The order of drugs is tabulated in order of most recent to oldest registration date.

No.	Generic Name of Drug	Brand Name and Company	First FDA/EMA Approved Date	Structure	Molecular Target	Route of Administration	Indication	Adverse Effects	Reference
1	Belantamab mafodotin-blmf	BLENREP GlaxoSmithKline, Brentford, England	FDA: 5 August 2020 EMA: 25 August 2020	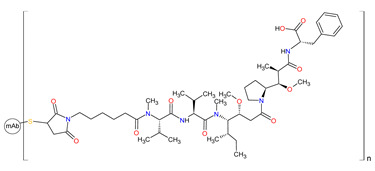	BCMA ^1^	Intravenous	Multiple Myeloma	Ocular toxicity, thrombocytopenia, infusion-related reactions, gastrointestinal disorders, pyrexia, fatigue	[265,266]
2	Polatuzumab vedotin-piiq	POLIVY Genentech, Inc., South San Francisco, CA, USA	FDA: 10 June 2019 EMA: 16 January 2020	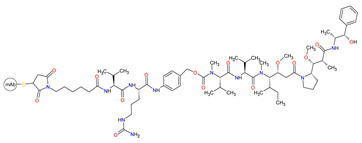	CD79b ^2^	Intravenous	Diffuse Large B-Cell Lymphoma	Cytopenias	[262,267,268]
3	Inotuzumab ozogamicin	BESPONSA Pfizer Inc., New York, NY, USA	FDA: 17 August 2017 EMA: 29 June 2017	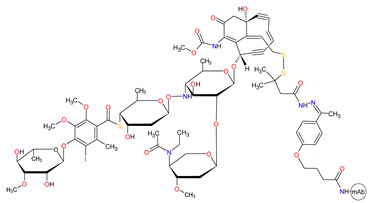	CD22 ^3^	Intravenous	Acute Lymphoblastic Leukemia	Cytopenias (including febrile neutropenia), infections, nausea, pyrexia, abnormal liver function and venoocclusive liver disease	[269,270]
4	Brentuximab vedotin	ADCETRIS Seattle Genetics, Inc., Bothell, WA, USA	FDA: 19 August 2011 EMA: 25 October 2012	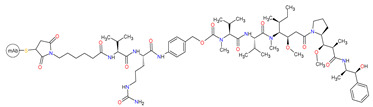	CD30 ^4^	Intravenous	Lymphoma, Hodgkin’s Lymphoma, Mycosis Fungoides	Neutropenia, anemia, peripheral sensory neuropathy, nausea, fatigue, constipation, diarrhea, vomiting, and pyrexia	[260,271,272]

^1^ BCMA: B-cell maturation antigen. ^2^ CD79b: cluster of differentiation 79b. ^3^ CD22: cluster of differentiation 22. ^4^ CD30: cluster of differentiation 30.

**Table 14:** Features of the immunotoxin drug approved by the Food and Drug Administration (FDA) from 2011 to 2021.

No.	Generic Name of Drug	Brand Name and Company	First FDA/EMA Approved Date	Class	Molecular Target	Route of Administration	Indication	Adverse Effects	Reference
1	Moxetumomab pasudotox-tdfk	LUMOXITI AstraZeneca, Cambridge, England	FDA: 13 September 2018 EMA: Not approved	Murine immunoglobulin and PE38 conjugate	CD22 ^1^	Intravenous	Hairy Cell Leukemia	Peripheral edema, nausea, fatigue, headache, pyrexia, decreased lymphocyte count, hemolytic uremic syndrome,	[276,277]

^1^ **CD22**: cluster of differentiation 22.

**Table 15:** Features of the enzymes drugs approved by the Food and Drug Administration (FDA) from 2011 to 2021. The order of drugs is tabulated in order of most recent to oldest registration date.

No.	Generic Name of Drug	Brand Name and Company	First FDA/EMA Approved Date	Class	Molecular Target	Route of Administration	Indication	Adverse Effects	Reference
1	Calaspargase pegol-mknl	ASPARLAS Servier Pharmaceuticals, Boston, MA, USA	FDA: 20 December 2018 EMA: Not approved	Asparagine-specific enzyme	Asparagine level	Intravenous	Acute Lymphoblastic Leukemia	Elevated transaminase, bilirubin increased, pancreatitis, abnormal clotting studies	[280]
2	Asparaginase *Erwinia chrysanthemi*	ERWINAZE Jazz Pharmaceuticals plc, Dublin, Ireland	FDA: 18 November 2011 EMA: nationally authorized	Asparagine-specific enzyme	Asparagine level	Intravenous	Acute Lymphoblastic Leukemia	Anaphylaxis, pancreatitis, abnormal transaminases, thrombosis, hemorrhage, nausea, vomiting, hyperglycemia.	[283,284,285]

**Table 16:** Features of the chimeric antigen receptor T-cells (CAR-T cells) drugs approved by the Food and Drug Administration (FDA) from 2011 to 2021. The order of drugs is tabulated in order of most recent to oldest registration date.

No.	Generic Name of Drug	Brand Name and Company	First FDA/EMA Approved Date	Class	Molecular Target	Route of Administration	Indication	Adverse Effects	Reference
1	Brexucabtagene autoleucel	TECARTUS Kite, a Gilead Company, Foster City, CA, USA	FDA: 24 July 2020 EMA: 14 December 2020	Genetically modified autologous T cells	CD19 ^1^	Intravenous	Mantle Cell Lymphoma	Cytokine release syndrome, cytopenias, hypotension, encephalopathy, fever, fatigue, tachycardia, arrhythmia, infection with pathogen unspecified, chills, hypoxia, cough, tremor, musculoskeletal pain, headache, nausea, edema, motor dysfunction, constipation, diarrhea, decreased appetite, dyspnea, rash, insomnia, pleural effusion, aphasia	[290,294,295]
2	Axicabtagene ciloleucel	YESCARTA Kite Pharma, Inc., Los Angeles, CA, USA	FDA: 18 October 2017 EMA: 23 August 2018	Genetically modified autologous T cells	CD19 ^1^	Intravenous	Large B-Cell Lymphoma, Follicular Lymphoma	Cytokine release syndrome, fever, hypotension, encephalopathy, tachycardia, fatigue, headache, febrile neutropenia, nausea, infections with pathogen unspecified, decreased appetite, chills, diarrhea, tremor, musculoskeletal pain, cough, hypoxia, constipation, vomiting, arrhythmias, dizziness	[296,297]
3	Tisagenlecleucel	KYMRIAH Novartis Pharmaceuticals Corporation, Basel, Switzerland	FDA: 30 August 2017 EMA: 23 August 2018	Genetically modified autologous T cells	CD19 ^1^	Intravenous	Acute Lymphoblastic Leukemia, Large B-Cell Lymphoma	Cytokine release syndrome, infections-pathogen unspecified, pyrexia, decreased appetite, hypogammaglobinemia, headache, encephalopathy, hypotension, bleeding, episodes, tachycardia, nausea, diarrhea, vomiting, viral infectious disorders, hypoxia, fatigue, acute kidney injury, edema, cough, delirium	[298,299]

^1^ **CD19**: cluster of differentiation 19.

## Data Availability

No new data were created or analyzed in this study. Data sharing is not applicable to this article.
